# Supplementary material for: Genetic Codes with No Dedicated Stop Codon: Context-Dependent Translation Termination
Source: Cell. 2016 Jul 28;166(3):691–702. doi: 10.1016/j.cell.2016.06.020 (PMC4967479; doi:10.1016/j.cell.2016.06.020)
Supplement: Document S1. Supplemental Experimental Procedures and Table S1 [file mmc1.pdf]

Cell, Volume *166*

Supplemental Information

Genetic Codes with No Dedicated Stop Codon:

Context-Dependent Translation Termination

Estienne Carl Swart, Valentina Serra, Giulio Petroni, and Mariusz Nowacki

Supplemental Experimental Procedures

Condylostoma magnum culturing

Condylostoma magnum strain COL2 (500-550 µm longest dimension) was isolated in 2007 as a single cell from rocky seaside pools near the Accademia Navale in Livorno, Italy. Cells were kept at room temperature and fed regularly with *Dunaliella tertiolecta* and occasionally with *Phaeodactylum tricornutum.* To grow *D. tertiolecta* and *P. tricornutum*, a saline solution was made with the following: 37 g of Red Sea Salt (company: Red Sea), 1 ml of Walne's solution (Walne, 1970) and 1 drop of multivitamin complex B (B1, B6, and B12; Bayer Benexol B12) made up to 1L with distilled water. Walne's solution was prepared as: 100 g $NaNO_3$, 45 g EDTA (disodium salt), 33.6 g H_3BO_3, 20 g $NaH_2PO_4.H_20$, 0.36 g $MnCl_2$, 1.8 g EDTA (disodium salt), 1 ml of TMS (Trace Metal Solution: 2.1 g $ZnCl_2$, 2.0 g $CoCl_2.6H_2O$, 0.9 g $(NH_4)_6Mo_7O_{24}.4H_2O$, 2.0 g $CuSO_4.5H_2O$, H_2O to 100 ml; acidified with a few drops of concentrated HCl to clarity), made up to 1 L in distilled water. The microalgal culture was incubated at 19°C with a 12 h light/dark cycle (Osram Daylight lamp, 36W/10 and Osram Fluora lamp, 40W/77).

Checks for potential contaminating transcripts

To check for possible contaminants (e.g. from the algal food source, *Pheodactylum tricornutum*) among the assembled *C. magnum* transcripts we examined sequence base composition and assembled rRNAs (MMETSP: CAMNT_0008266115, CAMNT_0008312561). Base composition is unimodal (mode 33% GC) and no rRNAs other than from *C. magnum* were found, suggesting that this data is predominantly comprised of *C. magnum* transcripts. *C. magnum*'s food source, the diatom *P. tricornutum* (European Nucleotide Archive (ENA): GCA_000150955.2), has transcripts that are more GC rich (mode 50% GC) than those of *C. magnum*, and has the standard genetic code (Data S1F). As evidenced by the absence of Pfam domains (Finn et al., 2014) matching typical mitochondrially-encoded ciliate proteins (e.g. COX1, COX2, COB, NAD5) during HMMER3 (Eddy, 2014) searches (default parameters, independent e-value < 1e-3) of the assembled *C. magnum* RNA-seq data, mitochondrial transcript levels are negligible.

Shotgun proteomics and verification of translation of UGA as tryptophan

200 µl of *C. magnum* cells (> 30,000 cells) were lysed in 1 ml of protein loading buffer (from a 10 ml stock of 2 ml 10% SDS; 1.2 ml 0.5 M Tris-Cl pH 6.8; 4.8 ml 50% glycerol; 1.2 mg Bromophenol blue; 500 µl β-Mercaptoethanol; 1.5 ml ddH_20) and stored at -20˚C until further processing. 5 µl of this sample was incubated at 95˚C for 5 minutes. SDS-PAGE was used to separate the proteins with a 5% stacking gel (2.2 ml ddH_20; 0.67 ml 30% acrylamide/Bis solution (Bio-Rad); 1 ml Tris-Cl (0.5 M, pH 6.8); 0.04 ml 10% SDS; 0.04 ammonium persulfate; 4 µl TEMED) on top of a 10% resolving gel (5.9 ml ddH_20; 5.0 ml 30% acrylamide/Bis solution (Bio-Rad); 3.8 ml Tris-Cl (0.5 M, pH 6.8); 0.15 ml 10% SDS; 0.15 ammonium persulfate; 6 µl TEMED) an electrophoresis buffer (3.2 g Tris; 14.4 g glycine; 1 g SDS made up to 1 L with ddH_20). After staining the gel with InstantBlue (Expedeon) and destaining in a 10% acetic acid, 25% methanol solution, 10 slices of ~1 mm width were cut from the resolving gel, and diced into six ~1 mm^3 cubes which were stored in 20% ethanol at -20˚C before further processing.

Gel cubes were washed with 50 mM Tris/HCl pH 8 (Tris buffer) and Tris buffer/acetonitrile (LC-MS grade, Fluka, Buchs, Switzerland) 50/50 before protein reduction with 50 mM DTT (Fluka, Buchs, Switzerland) in Tris buffer for 30 min at 37°C, and alkylation with 50 mM iodoacetamide (Fluka, Buchs, Switzerland) in Tris buffer for 30 min at 37°C in the dark. The gel cubes were then soaked with trypsin solution (10 ng/ml trypsin (Promega) in 20 mM Tris/HCl pH 8, 0.01% ProteaseMax (Promega)) for 30 min on ice, then covered by 5–10 ml 20 mM Tris/HCl before digestion for 60 min at 50°C. A 5 µl injection of the protein digest was then analyzed by liquid chromatography-tandem mass spectrometry (LC)-MS/MS (DIONEX Ultimate coupled to a QExactive mass spectrometer, ThermoFisher Scientific). Peptides were trapped on an Acclaim PepMap100 C18 pre-column (3 µm, 100 Å, 75 µm×2 cm, ThermoFisher Scientific, Reinach, Switzerland) and separated by backflush on a C18 column (5 µm, 100 Å, 75 µm×15 cm, Magic C18) by applying a 60 minute gradient of 5% to 40% acetonitrile in water and 0.1% formic acid, at a flow rate of 400 nl/min. The full-scan method was set with resolution at 70,000 with an automatic gain control (AGC) target of 1e06 and maximum ion injection time of 50 ms. The data-dependent method for precursor ion fragmentation was applied with the following settings: resolution 17,500, AGC of 1e05, maximum ion time of 110 milliseconds, mass window 2 *m*/*z*, collision energy 27, underfill ratio 1%, charge exclusion of unassigned and 1+ ions, and peptide match preferred, respectively. A database of six-frame translations of the *C. magnum* transcriptome assembly, translated with UAR=glutamine, UGA=tryptophan, was used as input for *in silico* peptide fragmentation and peptide identification by EasyProt (Gluck et al., 2013) (default parameters: 1% false discovery rate and two peptides for acceptance of a protein identification).

To verify tryptophan translation at UGA codons, we examined each of the peptides identified by mass spectrometry containing a tryptophan translated from a UGA codon (25 total; Data S1D). 22 of these peptides were in the same reading frame as the best BLASTX match of their transcript to *O. trifallax* predicted proteins (using the BLAST server at oxy.ciliate.org (Stover et al., 2012; Swart et al., 2013); e-value < 1e-6), and each of the remaining three peptides had no BLASTX matches to the transcript from which it was derived.

Codon usage prediction

To determine codon usage (Figure 1B and Figure S1) we extracted best BLASTX matching regions (-query_gencode 6; e-value < 1e-20) of coding sequences from poly(A) tailed MMETSP transcripts used as queries to an *O. trifallax* predicted protein database (Swart et al., 2013). For ciliates with the standard genetic code (e.g. the heterotrichs *Climacostomum virens* and *Fabrea salina*), our codon usage estimates for UAA, UAG and UGA codons are 0-0.008%, and appear to represent algorithmic errors (e.g. "stops" located close to the ends of BLAST matches, which may occur when the local alignment extends beyond true protein ends), and selenocysteine codons. Codon usage of the ciliates examined in this manuscript is provided as Data S1C.

Stop codon and 3' UTR identification

To predict the stops of *C. magnum* coding sequences in poly(A)-terminated transcripts (possessing a terminal poly(A) ≥ 7 nt), we visually inspected BLASTX (Camacho et al., 2009) results of *C. magnum* transcripts vs. proteins from *O. trifallax* (Swart et al., 2013) (local BLASTX; best BLASTX match; e-value < 1e-6; query genetic code 6); *T. thermophila* (Stover et al., 2012) and GenBank's nr (nonredundant) database were also used when there was uncertainty about the match ends from the *O. trifallax* BLASTX matches. The stop codon was chosen as the first UAA, UAG, or UGA codon downstream of the BLASTX match closest to the C-terminal end of the *O. trifallax* or *T. thermophila* proteins. Sequences where the top matches terminated close (~6 amino acids) to the predicted query stop codons were then selected. This procedure annotated 150 putative *C. magnum* 3' UTRs, and the coding sequence (CDS) regions upstream to the beginning of the best BLASTX matches (Data S1R). With the appropriate query genetic code and stop codons, this procedure was also used to annotate 50 3' UTRs from *B. japonicum*, *Climacostomum virens*, *Euplotes crassus* and *Pseudokeronopsis sp.,* and 70 3' UTRs from *Parduczia. sp.* (Data S1R). Note that UAA terminated coding sequences are underrepresented in the resultant *C. magnum* data set (0% of transcripts) relative to those automatically inferred from the Trinity transcriptome (11%) described in the next paragraph. In our inspection of *Parduczia sp.* transcripts (by means of BLASTX matches to ciliates and other eukaryotes) we found no examples of coding sequences terminated by either UAG or UAA as stops.

To generate larger data sets to analyze stop codons, Trinity (default parameters) was used to asssemble new transcriptomes from both the *C. magnum* and *Parduczia sp*. MMETSP RNA-seq data (Data S1X and Data S1Y, respectively). BLASTX searches (best matches; e-value < 1e-20; query genetic code 6) of poly(A)-tailed transcripts from *C. magnum* and *Parduczia sp.* vs. *O. trifallax* proteins were used to infer reading frame, excluding cases where the BLAST match was to the reverse complement of the strand possessing the poly(A). Poly(A) tails were trimmed down to 0, 1 or 2 nucleotides to maintain the reading frame when counting codons back from the transcript ends (position 0 in Figure 6; Figure S5 and S6). From these transcripts, single gene (Trinity classification), single isoform transcripts were selected for "stop" codon and ribo-seq analysis (yielding 1672 transcripts for *C. magnum* and 455 for *Parduczia sp.*; Data S1Z and S1AA).

Based on our analysis of the 3' UTR length distributions of the curated MMETSP transcripts, we generated a data set of transcripts with only a single possible stop in the region 60 nt upstream of the poly(A)-tailed Trinity assembled transcripts (excluding the poly(A) tail length), yielding 294 transcripts (Data S1AB). For *C. magnum* transcripts with only a single possible stop, the frequencies of UAG, UGA and UAA stops are 62%, 25% and 13%, respectively. Scanning downstream from 60 nt upstream of the poly(A) tail to identify the first downstream "stop" codon, i.e. the putative primary stop, 1378 (82%) transcripts have additional possible stop codons downstream of the putative primary stop. As judged from ribosome profiling data, this procedure correctly classifies the bulk of primary stop codons (with little readthrough; Figure 3D and Figure S3D-H). The overall length distribution of the 3' UTRs downstream of the Trinity transcript putative primary stops is similar to that of the manually curated 3' UTRs (peaking around 18-21 nt). 12 of 39 (31%) 3' UTRs with UAA as the primary stop are of length 0. For zero nt 3' UTRs, 8 of 12 are consistent with the positions of stops in other organisms, as judged by BLASTX searches, and/or by RPFs ending 11/12 nt downstream of the UAA (compared to 17 of the 27 3' UTRs > 0 nt long assessed by the same criteria); no evidence suggests that the remaining four zero nt 3' UTRs are incorrect predictions.

Stop codon readthrough detection and estimation

The fraction of readthrough, $r = cov_{stop} \div (cov_{stop} + cov_{downstream})$, is measured relative to the stop (positions +1 to +3) for transcripts with at least twenty 30 nt RPF 3' ends at positions +12 to +14 (the positions corresponding to the characteristic termination signal, as in Figure 3D). cov_{stop} = (30 nt RPF 3' end counts at positions +12 to +14) ÷ 3; $cov_{downstream}$ = (30 nt RPF 3' end counts from positions +17 to the 3' UTR end, excluding the poly(A) tail) ÷ (number of positions in 3' UTR at which 30 nt 3' RPF ends were counted). Note that due to the counting of only covered positions in the denominator of $cov_{downstream}$, readthrough will be overestimated (if all the positions in the 3' UTR were used instead, readthrough would be underestimated). Transcripts are considered to be read through if $r > 0$.

Stop codon usage estimation

For the 670 MMETSP transcriptomes we downloaded, stop codon usage was estimated for poly(A)-ending (≥ 7 nt) contigs from the MMETSP ESTScan (Iseli et al., 1999) coding sequence predictions (Keeling et al., 2014) ending on TAA, TAG, or TGA. Transcriptomes with ≥ 50 putative stop codons were used for stop codon usage estimation, excluding ciliates with non-standard

genetic codes except *B. japonicum*. Note that UAA stop codon usage is underestimated for transcriptomes with a significant proportion of non-ciliate transcripts (often originating from the sources indicated in Table S1). A table of stop codon counts for these transcriptomes is provided in Data S1W.

Sequence logos of regions flanking *C. magnum* sense and stop codons

Sequence logos were created with WebLogo 3.3 (Crooks et al., 2004). For 3' UTRs, sequence logos use a compositional adjustment of 3' UTR base frequencies, excluding the stop codon and poly(A) tail (e.g. for *C. magnum*: A=39%, C=5%, G=13%, U=43%). For coding sequence (CDS) positions immediately upstream of the stop, a compositional adjustment is done frame-wise for each of the three reading frames, based on manually curated coding sequence base frequencies (frame 1: A=32%, C=14%, G=33%, U=21%; frame 2: A=35%, C=19%, G=18%, U=28%; frame 3: A=31%, C=16%, G=19%, U=33%).

Multiple sequence alignments

MAFFT 7.017 (Katoh and Standley, 2013) was used for all multiple sequence alignments. The default parameters in Geneious 7.1.4 (Kearse et al., 2012) were used for both alignment methods.

d_N/d_S estimation

d_N/d_S values were estimated using codeml version 4.7b (Yang, 2007) for a pairwise alignment of *C. magnum* tryptophan-tRNA ligase (CAMNT_0008287141) and *Oxytricha* tryptophan-tRNA ligase (GenBank: EJY83191.1).

Genome sequencing, assembly and read mapping

A NucleoSpin Plant II kit (MACHEREY-NAGEL) was used to isolate total DNA from a 0.1 ml of *C. magnum* cells pelleted from a 2 L culture. We assembled a draft *C. magnum* macronuclear genome using the Minia genome assembler (Chikhi and Rizk, 2013) with default parameters and a k-mer size of 85. An additional assembly produced by the IDBA_UD assembler (Peng et al., 2012) was also examined in some cases (e.g. tRNA searches). Over the larger *C. magnum* contigs we examined, we observe reasonably even sequence coverage (mean ~140×). As is typical of ciliates, *C. magnum* has a micronuclear genome in addition to its macronuclear genome. In this assembly, micronuclear sequences are likely to be minimal since macronuclear DNA is typically highly amplified in large ciliates (> 1000×) (Prescott, 1994). We were also able to assemble a large portion (29.9 kb) of the *C. magnum* mitochondrial genome (contigs 3__len__11145, 0__len__6198, 1__len__11219 and, 2__len__1536; identified by BLAST searches vs. other ciliate mitochondrial genomes). Due to *C. magnum*'s unusual genetic code, an accurate automated gene prediction method still needs to be developed, and so we provide just the raw macronuclear genome assembly at present (European Nucleotide Archive accession: ERS696421). Paired-end reads were mapped to the draft *C. magnum* assembly using BWA (Li and Durbin, 2009) (default parameters).

Computational tRNA identification

tRNAscan-SE (Lowe and Eddy, 1997) with default parameters was initially used to predict tRNAs in the *C. magnum* Minia genome assembly. In our draft *C. magnum* genome assembly tRNAscan-SE did not detect a selenocysteine tRNA, but ARAGORN (default parameters) did (Figure S4F).

BLASTN searches (word size of 4) of the draft *C. magnum* genome detected no additional paralogs of $tRNA^{Trp}$(CCA) beyond those identified by tRNAscan-SE. No reads among those mapped to *C. magnum* $tRNA^{Trp}$(CCA) genes with STAR ((Dobin et al., 2013); default parameters) suggested the presence of unassembled sequences from undetected close tryptophan tRNA paralogs. ARAGORN (Laslett and Canback, 2004) searches (default parameters) found no *C. magnum* $tRNA^{Trp}$(UCA)'s at the read level, other than the mitochondrial and selenocysteine tRNAs (Figure S4A and S4F, respectively; Data S1H).

Reducing tRNAscan-SE's Cove cutoff score to 10 allowed the discovery of a single putative tRNA(UCA) with a Cove score of 11.51 (Figure S4D; Data S1G; on contig 14671__len__38937). This tRNA has an unusual eight base anticodon with a potential UCA anticodon complementary to the UGA codon and falls in a region with no mapped MMETSP RNA-seq reads (using STAR; Data S1I,J). For the same sequence plus one base, a leucine tRNA with a CAA anticodon is predicted by ARAGORN (Laslett and Canback, 2004) (Figure S4E; default parameters), making the anticodon recognized by the lower scoring tRNAscan-SE prediction doubtful. Only one other tRNA (Cove score 12.54) was found below the default scoring threshold of 20, and had no possible UCA anticodon. This tRNA also falls in a region with no mapped MMETSP RNA-seq reads (Data S1M,N). Expression of these candidate tRNAs is supported by tRNA-derived sRNA-seq reads, including reads with CCA tails characteristic of mature tRNAs (see next section; Data S1K,N).

sRNA-seq for tRNA identification and searches for $tRNA^{Trp}$(CCA) anticodon editing

Total RNA was isolated from > 1000 cells using an miRNAeasy Mini kit (Qiagen). 60-100 nt RNAs were size-selected on an electrophoretic gel and paired-end RNA-seq libraries (125 bp reads, only one direction was provided) were prepared using standard Illumina protocols by Fasteris (Geneva, Switzerland). After quality control and adaptor trimming by Fasteris there were 1.9 million 10-99 bp reads. Raw reads were deposited in the European Nucleotide Archive (accession numbers: ERS744875 and

ERS744876). 30-89 bp reads were mapped to the entire tRNA-encoding contigs using BWA (Li and Durbin, 2009) with default parameters.

To facilitate ease of viewing we extracted all the reads mapping to $tRNA^{Trp}$(CCA) genes in our assembly and mapped them back to a representative tRNA on contig 27450__len__809 with the Geneious read mapper and default maximum sensitivity parameters (Data S1P). None of the 164 reads mapping through or up to the first codon of the $tRNA^{Trp}$(CCA) anticodons had evidence of 1st position anticodon C→U editing (Data S1P,Q).

Searches for $tRNA^{Trp}$(CCA) anticodon editing by RT-PCR and Sanger sequencing
To search for CCA→UCA anticodon editing we performed either single cell RT-PCR (Trp1 and Trp2 primer combinations described later in this paragraph) or RT-PCR on RNA isolated from 15 *C. magnum* cells with the miRNAeasy Mini kit (Qiagen) in 30 ul of nuclease free water (Trp2_f and Trp2_rM primer). Single cells were isolated with a Gilson pipette and washed several times in saline water (33 g/L NaCl) followed by a single wash in distilled water. Individual cells in 2 ul of water, or 2 ul of purified RNA, were combined with 1 ul of reverse transcription primers, 1 ul dNTPs and 6 ul of nuclease free water. Cells were lysed at 65°C for 5 min. Reverse transcription was performed using Superscript III reverse transcriptase (Life Technologies). The following primers were used (primer names with "_r" suffixes were used for cDNA synthesis): Trp1_f: GGGGCTATAGCTCAGCGGAAG, Trp1_r: GTGAGGCTAGAGCGATTTGAACG, Trp2_f: GGGGCTATAGCTCAATGGTAGAG, Trp2_r: GTGAGGCTAGAGCGATTCGAAC, Trp2_rM: TGGTGAGGCTAGAGCGATTC. PCR products purified with the Wizard SV Gel and PCR Cleanup Kit (Promega) were cloned into pGEM-T easy vectors (Promega). Plasmids containing the RT-PCR products were isolated with the Wizard Plus SV Miniprep DNA Purification kit (Promega) before being Sanger sequenced by Microsynth (Switzerland).

In total 77 $tRNA^{Trp}$ sequences were obtained (20 - "Trp1" and 37 - "Trp2" and 20 – "Trp2M"; Data S1O). Just one of 77 sequenced clones had a C→T substitution at the 1st anticodon position (Data S1O,Q); however, since none of the 164 reads obtained from Illumina sRNA-seq had this substitution it seems more likely that it is an RTase or PCR error rather than a genuine editing event.

Detection of selenocysteine UGA codons
In Figure 6 and Figure S5 counts of a few UGA codons in selenogenes can be seen. The *C. magnum* and *Parduczia sp.* transcriptomes both encode selenogenes with the necessary SECIS (selenocysteine insertions sequence) elements for selenocysteine translation. As an example of these selenogenes, both transcriptomes encode a thioredoxin gene whose single catalytic selenocysteine is encoded by a UGA codon, just one codon upstream of its stop (also UGA; Figure S2E).

eRF1 phylogeny
To create an eRF1 phylogeny 16 representitive, manually annotated ciliate eRF1 protein sequences from MMETSP transcripts were chosen. Two additional predicted protein sequences for *Oxytricha trifallax* and *Tetrahymena thermophila*, were obtained from www.ciliate.org, and a human eRF1 protein sequence was obtained from UniProt (accession:P62495). *Carchesium polypinum* eRF1 was obtained from a manually annotated transcript from its Trinity transcriptome assembly. All the eRF1 sequences were then aligned using MAFFT (default parameters in Geneious 7.1.4). This alignment can be seen in Figure S7. To produce the phylogeny, a conserved block of 429 amino acids was manually selected as the input alignment for PhyML (Guindon et al., 2010) (substitution model LG, 100 bootstrap replicates, default parameters in Geneious 7.1.4).

Supplemental Tables

Table S1. Ciliate genetic codes. Related to Figure 1.

ID	"Stop" assignments			Class	Family	Bionomial name	Strain	Food/host	Contami -nation
	UAA	UAG	UGA						
MMETSP0127	*	*	*	Colopdea	Platyophryidae	Platyophrya macrostoma	WH	Bodo caudatus, Enterobacter aerogenes	yes
MMETSP1317	Q	Q	W/*	Karyorelictea	Geleiidae	Parduczia sp.	NA	?	no
MMETSP1395	*	*	W	Heterotrichea	Blepharismidae	Blepharisma japonicum	Stock R1072	bacteria	low
MMETSP1345	*	*	*	Heterotrichea	Climacostomidae	Fabrea salina	Unknown	bacteria	low
MMETSP1397	*	*	*	Heterotrichea	Climacostomidae	Climacostomum virens	Stock W-24	bacteria	no
MMETSP0210	Q/*	Q/*	W/*	Heterotrichea	Condylostomatidae	Condylostoma magnum	COL2	Phaeodactylum tricornutum	no
MMETSP0209	*	*	*	Litostomatea	Litonotidae	Litonotus pictus	P1	Euplotes crassus	no
MMETSP0467	Y	Y	*	Litostomatea	Mesodiniidae	Mesodinium pulex	SPMC105	Heterocapsa rotundata MMETSP0503	yes
MMETSP0798	Y	Y	*	Litostomatea	Mesodiniidae	Mesodinium rubra	CCMP2563	Geminigera cryophila	yes
MMETSP1018	Q	Q	*	Oligohymenophorea	Orchitophryidae	Anophryoides haemophila	AH6	lobster	no
MMETSP1019	Q	Q	*	Oligohymenophorea	Orchitophryidae	Anophryoides haemophila	AH6	lobster	low
MMETSP0125	Q	Q	*	Oligohymenophorea	Unknown	Aristerostoma sp.	ATCC 50986	Klebsiella, other bacteria	no
MMETSP0018	Q	Q	*	Oligohymenophorea	Uronematidae	Uronema sp.	Bbcil	?	no
MMETSP0472	Q	Q	*	Prostomatea	Colepidae	Tiarina fusus	LIS	Rhodomonas lens	yes
MMETSP0205	*	*	C	Spirotrichea	Euplotidae	Euplotes focardii	TN1	Dunaliella tertiolecta	low
MMETSP0206	*	*	C	Spirotrichea	Euplotidae	Euplotes focardii	TN1	Dunaliella tertiolecta	yes
MMETSP0213	*	*	C	Spirotrichea	Euplotidae	Euplotes harpa	FSP1.4	Dunaliella salina and Dunaliella tertiolecta	no
MMETSP1380	*	*	C	Spirotrichea	Euplotidae	Euplotes crassus	CT5	Dunaliella tertiolecta	low
MMETSP0216	*	*	*	Spirotrichea	Protocruziidae	Protocruzia adherens	Boccale	Dunaliella tertiolecta	no
MMETSP0211	Q	Q	*	Spirotrichea	Pseudokeronopsidae	Pseudokeronopsis sp.	OXSARD2	Phaeodactylum tricornutum	?
MMETSP1396	Q	Q	*	Spirotrichea	Pseudokeronopsidae	Pseudokeronopsis sp.	Brazil	bacteria, Phaeodactylum tricornutum	no
MMETSP0123	Q	Q	*	Spirotrichea	Ptychocylididae	Favella ehrenbergii	Fehren 1	Heterocapsa triquetra, Mantoniella squamata, Isochrysis galbana	no
MMETSP0434	Q	Q	*	Spirotrichea	Ptychocylididae	Favella taraikaensis	Fe Narragansett Bay	Heterosigma akashiwo CCMP3107	no
MMETSP0436	Q	Q	*	Spirotrichea	Ptychocylididae	Favella taraikaensis	Fe Narragansett Bay	Heterocapsa triquetra, CCMP 448	no
MMETSP0208	Q	Q	*	Spirotrichea	Strombidiidae	Strombidium inclinatum	S3	Dunaliella tertiolecta	no
MMETSP0449	Q	Q	*	Spirotrichea	Strombidiidae	Strombidium rassoulzadegani	ras09	Tetraselmis chui PLY429	no
MMETSP0126	Q	Q	*	Spirotrichea	Strombidinopsidae	Strombidinopsis acuminatum	SPMC142	Heterocapsa triquetra, Rhodomonas sp. (CCMP 755), Mantoniella squamata, Isochrysis galbana	yes
MMETSP0463	Q	Q	*	Spirotrichea	Strombidinopsidae	Strombidinopsis sp.	SopsisLIS2011	Rhodomonas lens	low

Supplemental References

Camacho, C., Coulouris, G., Avagyan, V., Ma, N., Papadopoulos, J., Bealer, K., and Madden, T.L. (2009). BLAST+: architecture and applications. BMC bioinformatics *10*, 421.

Chikhi, R., and Rizk, G. (2013). Space-efficient and exact de Bruijn graph representation based on a Bloom filter. Algorithms for molecular biology : AMB *8*, 22.

Crooks, G.E., Hon, G., Chandonia, J.M., and Brenner, S.E. (2004). WebLogo: a sequence logo generator. Genome research *14*, 1188-1190.

Dobin, A., Davis, C.A., Schlesinger, F., Drenkow, J., Zaleski, C., Jha, S., Batut, P., Chaisson, M., and Gingeras, T.R. (2013). STAR: ultrafast universal RNA-seq aligner. Bioinformatics *29*, 15-21.

Finn, R.D., Bateman, A., Clements, J., Coggill, P., Eberhardt, R.Y., Eddy, S.R., Heger, A., Hetherington, K., Holm, L., Mistry, J.*, et al.* (2014). Pfam: the protein families database. Nucleic acids research *42*, D222-230.

Gluck, F., Hoogland, C., Antinori, P., Robin, X., Nikitin, F., Zufferey, A., Pasquarello, C., Fetaud, V., Dayon, L., Muller, M.*, et al.* (2013). EasyProt--an easy-to-use graphical platform for proteomics data analysis. Journal of proteomics *79*, 146-160.

Guindon, S., Dufayard, J.F., Lefort, V., Anisimova, M., Hordijk, W., and Gascuel, O. (2010). New algorithms and methods to estimate maximum-likelihood phylogenies: assessing the performance of PhyML 3.0. Systematic biology *59*, 307-321.

Iseli, C., Jongeneel, C.V., and Bucher, P. (1999). ESTScan: a program for detecting, evaluating, and reconstructing potential coding regions in EST sequences. Proceedings / International Conference on Intelligent Systems for Molecular Biology ; ISMB International Conference on Intelligent Systems for Molecular Biology, 138-148.

Katoh, K., and Standley, D.M. (2013). MAFFT multiple sequence alignment software version 7: improvements in performance and usability. Molecular biology and evolution *30*, 772-780.

Kearse, M., Moir, R., Wilson, A., Stones-Havas, S., Cheung, M., Sturrock, S., Buxton, S., Cooper, A., Markowitz, S., Duran, C.*, et al.* (2012). Geneious Basic: an integrated and extendable desktop software platform for the organization and analysis of sequence data. Bioinformatics *28*, 1647-1649.

Laslett, D., and Canback, B. (2004). ARAGORN, a program to detect tRNA genes and tmRNA genes in nucleotide sequences. Nucleic acids research *32*, 11-16.

Li, H., and Durbin, R. (2009). Fast and accurate short read alignment with Burrows-Wheeler transform. Bioinformatics *25*, 1754-1760.

Lowe, T.M., and Eddy, S.R. (1997). tRNAscan-SE: a program for improved detection of transfer RNA genes in genomic sequence. Nucleic acids research *25*, 955-964.

Peng, Y., Leung, H.C., Yiu, S.M., and Chin, F.Y. (2012). IDBA-UD: a de novo assembler for single-cell and metagenomic sequencing data with highly uneven depth. Bioinformatics *28*, 1420-1428.

Prescott, D.M. (1994). The DNA of ciliated protozoa. Microbiological reviews *58*, 233-267.

Stover, N.A., Punia, R.S., Bowen, M.S., Dolins, S.B., and Clark, T.G. (2012). Tetrahymena Genome Database Wiki: a community-maintained model organism database. Database : the journal of biological databases and curation *2012*, bas007.

Walne, P.R. (1970). Studies on the food value of nineteen genera of algae to juvenile bivalves of the genera Ostrea, Crassostrea, Mercenaria and Mytilus (London,: H.M.S.O.).

Yang, Z. (2007). PAML 4: phylogenetic analysis by maximum likelihood. Molecular biology and evolution *24*, 1586-1591.
