# Supplementary material for: Genetic Codes with No Dedicated Stop Codon: Context-Dependent Translation Termination
Source: Cell. 2016 Jul 28;166(3):691–702. doi: 10.1016/j.cell.2016.06.020 (PMC4967479; doi:10.1016/j.cell.2016.06.020)
Supplement: Data S1. Supporting Data, Related to the Experimental Procedures — (A) genetic_code_predictions_ciliates.tar.gz: Genetic code predictions for 27 MMETSP ciliates and the peritrichous ciliates Carchesium polypinum and Campanella umbellaria. For the sequence logos the codons underlying positions 1-64 are (in order): TTT, TTC, TTA, TTG, TCT, TCC, TCA, TCG, TAT, TAC, TAA, TAG, TGT, TGC, TGA, TGG, CTT, CTC, CTA, CTG, CCT, CCC, CCA, CCG, CAT, CAC, CAA, CAG, CGT, CGC, CGA, CGG, ATT, ATC, ATA, ATG, ACT, ACC, ACA, ACG, AAT, AAC, AAA, AAG, AGT, AGC, AGA, AGG, GTT, GTC, GTA, GTG, GCT, GCC, GCA, GCG, GAT, GAC, GAA, GAG, GGT, GGC, GGA, GGG. (B) Genetic_code_predictions_MMETSP_nonciliates.tar.gz: Genetic code predictions for 636 MMETSP non-ciliate eukaryotes. Sequence logo codons as previous. (C) ciliate_codon_usage.txt. Ciliate codon usage. (D) peptides_confirming_W-UGA.txt: Peptide sequences from mass spectrometry confirming incorporation of tryptophan into C. magnum proteins. (E) mass-spec_run.xls: Summary of EasyProt analysis of mass spectrometry data. (F) P_tricornutum.genetic_code_prediction.pdf: Genetic code prediction and codon usage for P. tricornutum. (G) minia_asm_k.85.tRNAscan-SE-cove10.txt: tRNAscan-SE tRNA predictions for main C. magnum genome assembly. (H) tRNA_UCA_raw_reads_aragorn.txt: ARAGORN tRNA predictions wth UCA anticodons in raw genomic DNA sequence data. (I) 14671__len__38937_tRNA_UCA.sam.gz: MMETSP RNA-seq reads mapped to contig 14671__len__38937. (J) 14671__len__38937_tRNA_UCA.gff: tRNA and surrounding CDS annotation for 14671__len__38937. (K) 14671__len__38937.30-89nt.sam.gz: sRNA-seq data (30-89 nt) mapping to contig 14671__len__38937. (L) 1397__len__25408.sam.gz: MMETSP RNA-seq reads mapped to contig 1397__len__25408. (M) 1397__len__25408.gff: tRNA annotation for contig 1397__len_25408. (N) 1397__len__25408.30-89nt.sam.gz: sRNA-seq data (30-89 nt) mapping to contig 1397__len__25408. (O) 77_Sanger_seqs_to_tRNA-Trp_27450__len__809.sam.gz: Sanger sequences for clones of RT-PCR targeting tRNA-Trp(CCA). See ‘Q’ for associ [file mmc2.zip › supp_data/P_tricornutum_V1_genetic_code_prediction.pdf]

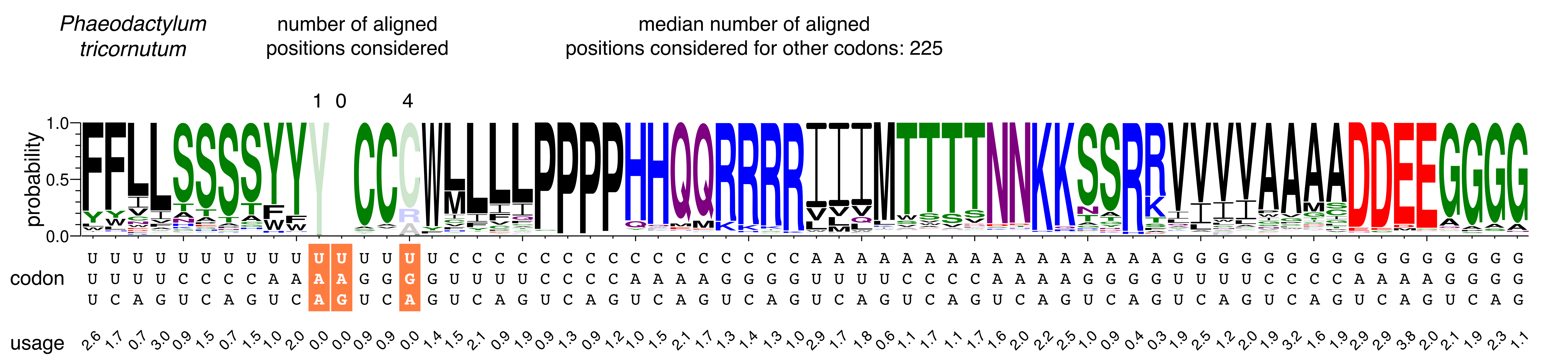

Phaeodactylum tricornutum
number of aligned positions considered
median number of aligned positions considered for other codons: 225
1 0 4
probability
1.0
0.5
0.0
codon
U U U U U U U U U U U U U U U U C C C C C C C C C C C C C C C C A A A A A A A A A A A A A A A A G G G G G G G G G G G G G G G G
U U U U C C C C A A A A G G G G U U U U C C C C A A A A G G G G U U U U C C C C A A A A G G G G U U U U C C C C A A A A G G G G
U C A G U C A G U C A G U C A G U C A G U C A G U C A G U C A G U C A G U C A G U C A G U C A G U C A G U C A G U C A G U C A G
usage
2.6 1.7 0.7 3.0 0.9 1.5 0.7 1.5 1.0 2.0 0.0 0.0 0.9 0.9 0.0 1.4 1.5 2.1 0.9 1.9 0.9 1.3 0.9 1.2 1.0 1.5 2.1 1.7 1.3 1.4 1.3 1.0 2.9 1.7 1.8 0.6 1.1 1.7 1.1 1.7 1.6 2.0 2.2 2.5 1.0 0.9 0.4 0.3 1.9 2.5 1.2 2.0 1.9 3.2 1.6 1.9 2.9 2.9 3.8 2.0 2.1 1.9 2.3 1.1
