# Supplementary figures and images for: Genetic Codes with No Dedicated Stop Codon: Context-Dependent Translation Termination
Source: Cell. 2016 Jul 28;166(3):691–702. doi: 10.1016/j.cell.2016.06.020 (PMC4967479; doi:10.1016/j.cell.2016.06.020)

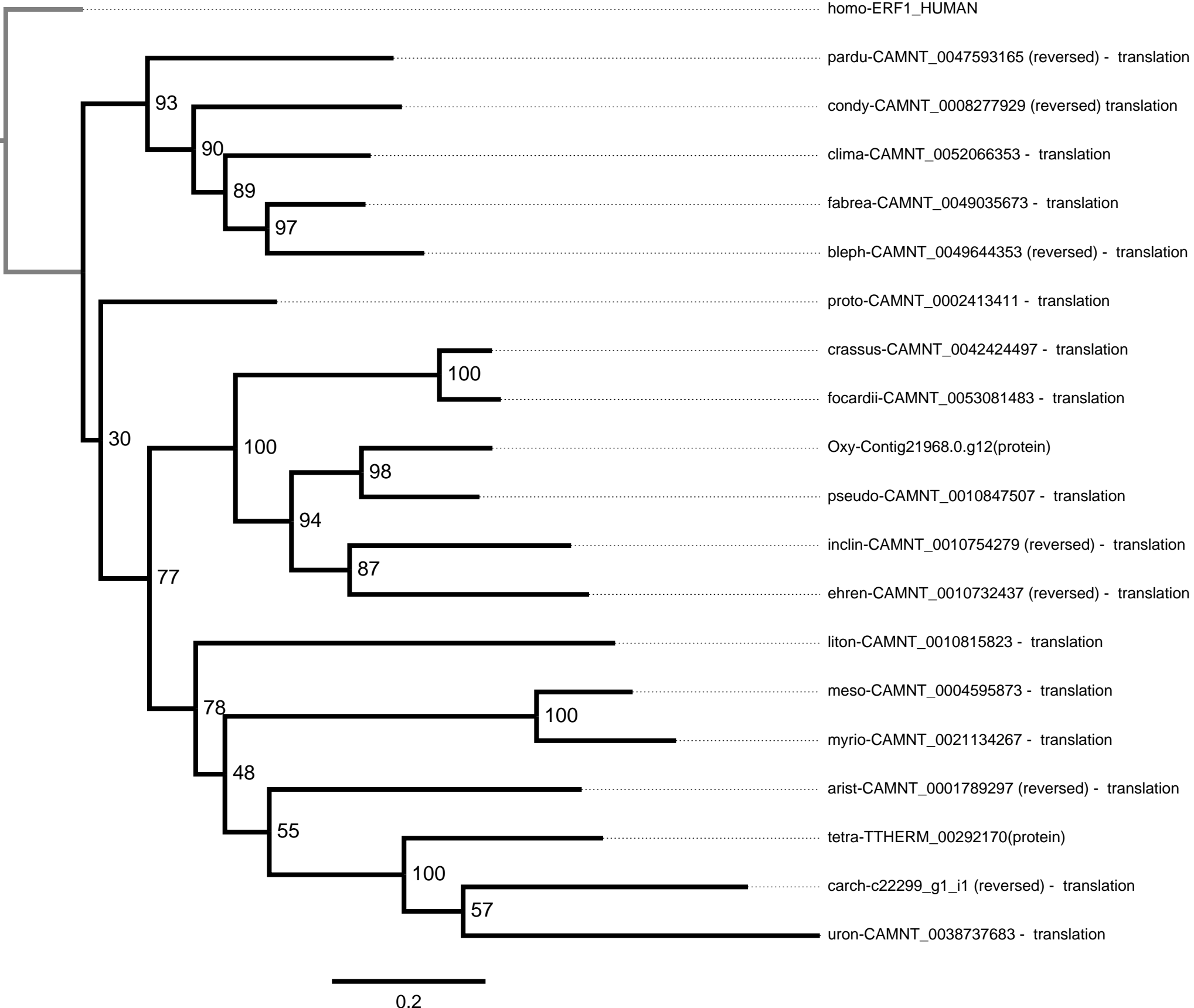

Supplement: Data S1. Supporting Data, Related to the Experimental Procedures — (A) genetic_code_predictions_ciliates.tar.gz: Genetic code predictions for 27 MMETSP ciliates and the peritrichous ciliates Carchesium polypinum and Campanella umbellaria. For the sequence logos the codons underlying positions 1-64 are (in order): TTT, TTC, TTA, TTG, TCT, TCC, TCA, TCG, TAT, TAC, TAA, TAG, TGT, TGC, TGA, TGG, CTT, CTC, CTA, CTG, CCT, CCC, CCA, CCG, CAT, CAC, CAA, CAG, CGT, CGC, CGA, CGG, ATT, ATC, ATA, ATG, ACT, ACC, ACA, ACG, AAT, AAC, AAA, AAG, AGT, AGC, AGA, AGG, GTT, GTC, GTA, GTG, GCT, GCC, GCA, GCG, GAT, GAC, GAA, GAG, GGT, GGC, GGA, GGG. (B) Genetic_code_predictions_MMETSP_nonciliates.tar.gz: Genetic code predictions for 636 MMETSP non-ciliate eukaryotes. Sequence logo codons as previous. (C) ciliate_codon_usage.txt. Ciliate codon usage. (D) peptides_confirming_W-UGA.txt: Peptide sequences from mass spectrometry confirming incorporation of tryptophan into C. magnum proteins. (E) mass-spec_run.xls: Summary of EasyProt analysis of mass spectrometry data. (F) P_tricornutum.genetic_code_prediction.pdf: Genetic code prediction and codon usage for P. tricornutum. (G) minia_asm_k.85.tRNAscan-SE-cove10.txt: tRNAscan-SE tRNA predictions for main C. magnum genome assembly. (H) tRNA_UCA_raw_reads_aragorn.txt: ARAGORN tRNA predictions wth UCA anticodons in raw genomic DNA sequence data. (I) 14671__len__38937_tRNA_UCA.sam.gz: MMETSP RNA-seq reads mapped to contig 14671__len__38937. (J) 14671__len__38937_tRNA_UCA.gff: tRNA and surrounding CDS annotation for 14671__len__38937. (K) 14671__len__38937.30-89nt.sam.gz: sRNA-seq data (30-89 nt) mapping to contig 14671__len__38937. (L) 1397__len__25408.sam.gz: MMETSP RNA-seq reads mapped to contig 1397__len__25408. (M) 1397__len__25408.gff: tRNA annotation for contig 1397__len_25408. (N) 1397__len__25408.30-89nt.sam.gz: sRNA-seq data (30-89 nt) mapping to contig 1397__len__25408. (O) 77_Sanger_seqs_to_tRNA-Trp_27450__len__809.sam.gz: Sanger sequences for clones of RT-PCR targeting tRNA-Trp(CCA). See ‘Q’ for associ [file mmc2.zip › supp_data/ML_tree_final_alignment_select_ciliates_V1.pdf]
