# Supplementary material for: Genetic Codes with No Dedicated Stop Codon: Context-Dependent Translation Termination
Source: Cell. 2016 Jul 28;166(3):691–702. doi: 10.1016/j.cell.2016.06.020 (PMC4967479; doi:10.1016/j.cell.2016.06.020)
Supplement: Document S2. Article plus Supplemental Information [file mmc3.pdf]

Cell

Article

Genetic Codes with No Dedicated Stop Codon: Context-Dependent Translation Termination

Graphical Abstract

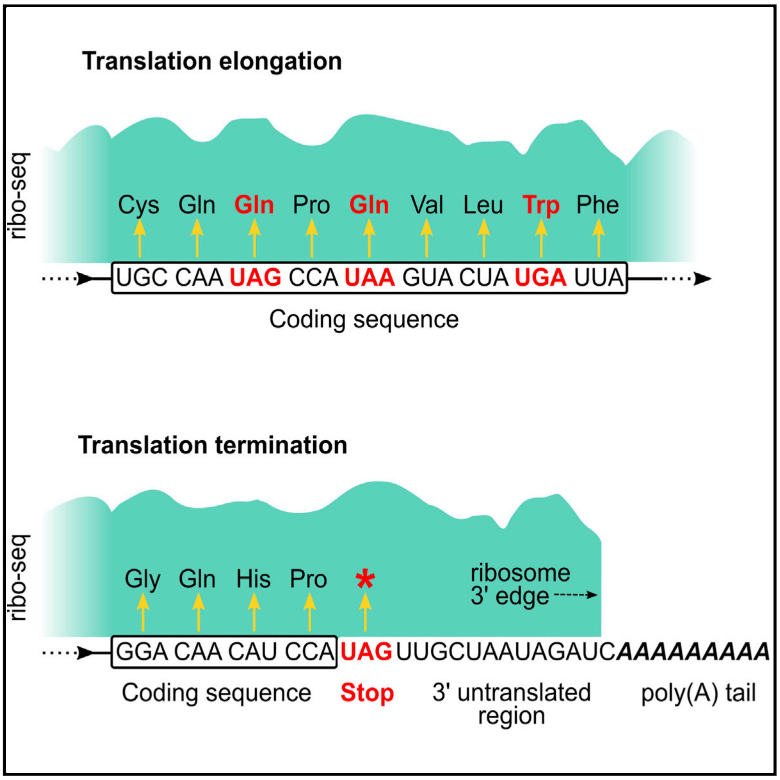

Authors

Estienne Carl Swart, Valentina Serra, Giulio Petroni, Mariusz Nowacki

Correspondence

mariusz.nowacki@izb.unibe.ch

In Brief

In some ciliates, all three "stop codons" can either terminate translation or code for an amino acid. Ribosomes may interpret this ambiguity using downstream features in the transcript, indicating that translational termination can be context-dependent.

Highlights

- Alternative nuclear genetic codes continue to be discovered in ciliates
- Genetic codes with stops and all their codons encoding standard amino acids exist
- Transcript ends may distinguish stop codons as such in ambiguous genetic codes
- The ability to resolve genetic code ambiguity may enable genetic code evolution

Swart et al., 2016, Cell *166*, 691–702
July 28, 2016 © 2016 The Author(s). Published by Elsevier Inc.
http://dx.doi.org/10.1016/j.cell.2016.06.020

CellPress

Cell

Genetic Codes with No Dedicated Stop Codon: Context-Dependent Translation Termination

Estienne Carl Swart,[1] Valentina Serra,[2] Giulio Petroni,[2] and Mariusz Nowacki[1,*]
[1]Institute of Cell Biology, University of Bern, 3012 Bern, Switzerland
[2]Department of Biology, University of Pisa, Pisa 56126, Italy
*Correspondence: mariusz.nowacki@izb.unibe.ch
http://dx.doi.org/10.1016/j.cell.2016.06.020

SUMMARY

The prevailing view of the nuclear genetic code is that it is largely frozen and unambiguous. Flexibility in the nuclear genetic code has been demonstrated in ciliates that reassign standard stop codons to amino acids, resulting in seven variant genetic codes, including three previously undescribed ones reported here. Surprisingly, in two of these species, we find efficient translation of all 64 codons as standard amino acids and recognition of either one or all three stop codons. How, therefore, does the translation machinery interpret a "stop" codon? We provide evidence, based on ribosomal profiling and "stop" codon depletion shortly before coding sequence ends, that mRNA 3′ ends may contribute to distinguishing stop from sense in a context-dependent manner. We further propose that such context-dependent termination/readthrough suppression near transcript ends enables genetic code evolution.

INTRODUCTION

The first exceptions to the supposed universality of eukaryotic nuclear genetic codes were reported in ciliates (Caron and Meyer, 1985; Helftenbein, 1985; Horowitz and Gorovsky, 1985; Preer et al., 1985). Subsequently, additional genetic codes were discovered in other ciliates, all due to stop codon reassignments, and appear to recur independently in different ciliate lineages (Lozupone et al., 2001; Sánchez-Silva et al., 2003; Tourancheau et al., 1995). Genetic code evolution is considered to have both an ancient phase, which gave rise to the standard genetic code before the radiation of bacteria, archaea, and eukaryotes, and a modern phase, which led to diversification from the standard code (Sengupta and Higgs, 2015). Thus far, alternative nuclear genetic codes have only been found in three major eukaryotic lineages other than ciliates. The first alternative nuclear genetic code, discovered in ciliates, with the UAA and UAG stop codons reassigned to glutamine, is also present in green algae (*Acetabularia* and *Batophora*) (Schneider and de Groot, 1991; Schneider et al., 1989) and diplomonads (Keeling and Doolittle, 1996). Alternative nuclear genetic codes, with CUG reassigned from leucine, also occur in the yeasts *Candida albicans* (predominantly to serine) and *Pachysolen tannophilus* (to alanine) (Gomes et al., 2007; Mühlhausen et al., 2016; Santos and Tuite, 1995).

Other than the diversity of genetic codes in ciliates, the greatest number of variant genetic codes are found in mitochondria (Knight et al., 2001), whose diversification may have been facilitated by their small genomes and strong mutational biases, which increase the likelihood of loss and reassignment of rare codons (Osawa and Jukes, 1989). Expressed ciliate genomes (macronuclear genomes) are not especially small (typically 50–100 Mb) (Swart et al., 2013), and the manner in which changes in their genetic codes arose may not be as straightforward as that in smaller mitochondrial genomes. Alternative explanations for the evolution of ciliate genetic codes, such as the abolishment of recognition of certain stop codons by mutations in the stop-recognizing translation termination factor eukaryotic release factor 1 (eRF1) allowing codon reassignment have therefore been proposed (Lozupone et al., 2001).

While the genetic code is classically taught as being unambiguous, and indeed may largely be so, we now know this is an oversimplification. Since the original discovery of the standard genetic code, alternative translational interpretations of codons have been found, most notably in the use of the UGA codon for selenocysteine incorporation, in the context of special mRNA stem-loops in the UTRs of a small number of protein-coding genes (Nasim et al., 2000). An additional form of codon ambiguity, translational readthrough of stop codons, is now also recognized as pervasive, but usually weak, in eukaryotes, occurring at a few percent or less compared to the non-readthrough form (e.g., Dunn et al., 2013; Harrell et al., 2002; Roy et al., 2015). Translational readthrough usually gives rise to short protein extensions, e.g., a median length of 35 amino acids in *Drosophila* (Jungreis et al., 2011). Readthrough is enabled by near-cognate pairing of tRNAs to codons, with either the first or third anticodon base non-canonically paired (Blanchet et al., 2014). Thus, there is competition for the same codons between eRF1 and tRNAs.

Although the options for engineering of new genetic codes with artificial amino acids have been proliferating (Lemke, 2014), many important questions about natural genetic codes remain unresolved. Among these questions, are basic ones of how codons are recognized in variant genetic codes with stop codon reassignments and whether there is competition between eRF1 and stop-cognate tRNAs for the same codons. Experimental evidence attempting to address the former problem has been conflicting, supporting either loss or ongoing recognition of reassigned stop codons by eRF1 (Eliseev et al., 2011; Lekomtsev et al., 2007; Salas-Marco et al., 2006; Vallabhaneni et al., 2009).

This is an open access article under the CC BY license (http://creativecommons.org/licenses/by/4.0/).

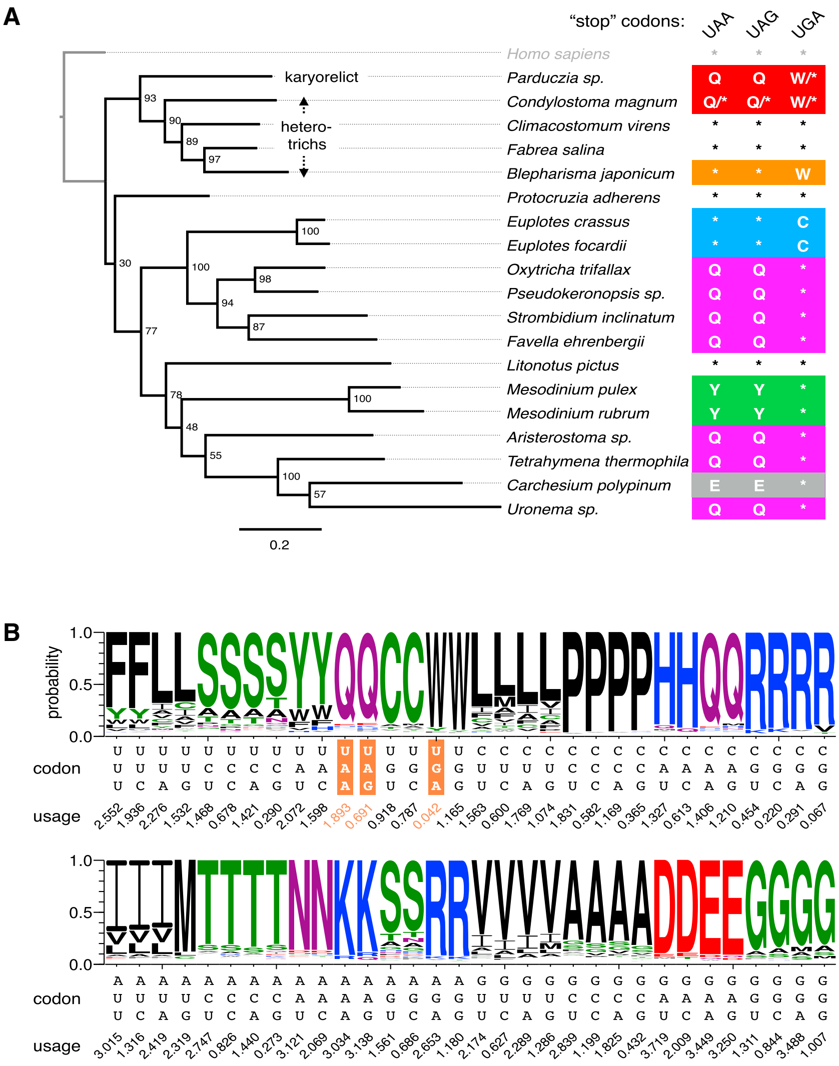

Figure 1. New Genetic Codes

(A) Stop codon reassignments (Q, glutamine; W, tryptophan; C, cysteine; Y, tyrosine; *, stop) are mapped onto an eRF1 maximum likelihood phylogeny. *Homo sapiens* (standard genetic code) is an outgroup. Bootstrap support for every node is shown. Scale bar indicates amino acid substitutions per site. UGA codons were previously found in the coding sequences of *Blepharisma americanum* and were predicted to encode tryptophan (Eliseev et al., 2011; Lozupone et al., 2001). Experimental assays in *Blepharisma japonicum* suggest its eRF1 recognizes all three standard stop codons (Eliseev et al., 2011). It should be noted that ciliates from the family Mesodiniidae have both a unique genetic code (UAG/UAA = UAR = tyrosine; UGA = stop) and extremely divergent rRNAs (Johnson et al., 2004).

(B) Predicted *C. magnum* genetic code. Stop codons are highlighted in orange. Predicted amino acids are those with maximal heights. Codon usage inferred from translated BLAST matches is shown below the codons. UAA and UAG codons were previously predicted to encode glutamine (Lozupone et al., 2001; Tourancheau et al., 1995).

See also Figure S1 and Table S1.

With extensive sequence data spanning a wide range of eukaryotes, including ciliates, now available, uncertain genetic codes may be properly determined, and consequently, the proposed basis for nuclear genetic code diversification is also ripe for reinvestigation. We present the new genetic codes we discovered in the course of screening a large collection of eukaryotic transcriptomes, how codons may have multiple meanings in two of these codes, and the consequences of tolerance of genetic code ambiguity for genetic code evolution.

RESULTS

Genetic Codes in which All 64 Codons Encode Standard Amino Acids

To identify and classify reassigned codons, we used a computational screening approach to search the Marine Microbial Eukaryote Transcriptome Sequencing Project (MMETSP) transcriptomes (Keeling et al., 2014). We found that like *Bembidion americanum*, *Bradyrhizobium japonicum* uses UGA as a tryptophan codon, although it does so at low levels (0.059%) and hence this reassignment may easily go undetected in small sequence samples (Figures 1B, S1A, and S1B). Thus, given this reassignment and previous experimental results (Eliseev et al., 2011), we deduce that *B. japonicum*'s eRF1 and at least one of its tryptophan tRNAs may be in competition for the same codon.

Because MMETSP represents the current broadest eukaryotic molecular diversity survey (Keeling et al., 2014) we screened all its transcriptomes to search for new genetic codes. In our screen, we discovered three new genetic codes among 24 ciliate species (Figures 1A, 1B and S1; Data S1A), but no new codes in the remaining 265 eukaryotes (Data S1B). Unexpectedly, in two of these genetic codes, belonging to the heterotrichous ciliate *Condylostoma magnum* and an unclassified karyorelict (18S rRNA 95% identical to that of *Parduzcia orbis* [Edgcomb et al., 2011]; *Parduzcia sp.* hereafter) all three "stop" codons are predicted to be reassigned to amino acids: UAA = Q, UAG = Q, UGA = W. As the remaining *C. magnum* and *Parduzcia sp.* codons encode standard amino acids (Figures 1A and S1A), all 64 of their codons are translated. Hence, the question is if and how translation termination occurs given these codes.

Because the UGA codon usage in *C. magnum*, *Parduzcia sp.*, and *B. japonicum* is relatively low (0.042%, 0.120%, and 0.059%, respectively), to computationally assess the hypothesis that the *C. magnum* and *Parduzcia sp*. genes with in-frame UGA codons are functional, and not simply pseudogenes with in frame

stops, we sought essential single copy genes with in-frame UGAs and examined their substitution rates. In-frame UGA codons are present in critical genes, such as *C. magnum* tryptophan-tRNA ligase (Figure 2B; MMETSP0210: CAMNT_0008287141) and eRF1 of *Parduczia sp.* (MMETSP1317: CAMNT_0047593165). Substitution rates of genes such as these support the hypothesis of functionality since they indicate strong purifying selection, e.g., for *C. magnum* tryptophan-tRNA ligase aligned to *Oxytricha trifallax* tryptophan-tRNA ligase, d_N/d_S is 0.013 (d_N/d_S = nonsynonymous substitutions per nonsynonymous site over synonymous substitutions per synonymous site; d_N/d_S <1 indicates purifying selection) (Yang, 2007). The hypothesis that UGA codons are translated was assessed experimentally in two ways: we determined that UGA codons are translated as tryptophan by protein mass spectrometry (Data S1D and S1E); using ribosome profiling we observe that ribosomes efficiently translate through UGA codons, as they also do through UAG and UAA codons (Figures 2B and S3E).

The Genetic Codes of *C. magnum* and *Parduczia sp.* Are Ambiguous

Given evidence that all three "stop" codons in the *C. magnum* and *Parduczia sp.* genetic codes can be translated, we wished to assess how translation termination occurs. To investigate the nature of translation termination in *C. magnum* and *Parduczia sp.* we began by examining histone H4 coding sequence ends, since the proteins encoded by these sequences are among the most highly conserved proteins and typically have the same C-terminal residues (e.g., 95% of 105 reviewed UniProt histone H4 proteins end with two glycines; Feb 9, 2015). With respect to the conserved C-terminal amino acid of histone H4 homologs in other eukaryotes, each of the *C. magnum* histone H4 paralog coding sequences is expected to end with a C-terminal glycine codon (Figure 2C). The codon immediately following this, either UAG or UGA, is therefore a candidate stop. The coding sequence of the single histone H4 in the *Parduczia sp.* transcriptome is followed by a UGA codon at the expected stop position (Figure 2C). With respect to aligned homologs from other organisms, all the *Parduczia sp.* transcripts we inspected have a UGA where a stop codon would normally be expected. *C. magnum* also has transcripts that have only the possibility of UAA stops in proximity to where stops are expected (Figures S2B–S2D). From the sequence alignments, we therefore infer that *C. magnum*'s eRF1 recognizes all three standard stop codons and hence needs to outcompete stop cognate tRNAs to terminate translation.

To test whether translation termination occurs at the putative histone H4 stop codons, we used ribosome profiling (ribo-seq). For *C. magnum*'s histone H4.1b and H4.1c forms, it can be seen that translation terminates precisely at the predicted stop codons (Figure 2D), whereas it does so with a small amount of imprecision for H4.1d (Figure 3A; H4.1a was insufficiently covered by ribo-seq reads to assess termination). In general, translation terminating *C. magnum* translation terminating ribosome-protected fragments (RPFs) end 11/12 nucleotides (nt) after stop codon 3′ nt (Figure 3D—compare to sense codons in Figure 3C; Figure 2D is a typical example). Consequently, both the primary and secondary H4.1d stop codons, UAG and UAA, trigger translation termination, and the typical histone H4 C-terminus may occasionally be extended by one or more amino acids.

While readthrough is conventionally classified as translation of stop codons by near-cognate tRNAs, in *C. magnum*, which has stop cognate tRNAs (see next section), translation through stop codons by near-cognate tRNAs is effectively indistinguishable from translation by cognate tRNAs in ribo-seq data. Therefore, for the sake of simplicity, in *C. magnum*, we classify readthrough as translation through codons that typically trigger translation termination (as for H4.1d). It should be noted that in *C. magnum*, multiple translation termination opportunities often exist before the ribosome translates into poly(A) tails (on average approximately five codons intervene between the primary and additional downstream non-primary stops). As a consequence, if extensions result from readthrough they are typically expected to be very short. Even though multiple possible stop codons exist, examples of imprecise termination as in H4.1d are in the minority: ~90% of transcripts examined with >20 RPFs situated at their stops show no readthrough. Thus, overall readthrough is quite low, e.g., a mean of <1.8% and median of 0% (Figure S3K). The small amount of readthrough that does occur is most readily detected when the ribosome occupies downstream stops (Figure 3E).

Multiple lines of evidence therefore demonstrate that "stop" codons as a class in the *C. magnum* and *Parduczia sp.* genetic codes are ambiguous, whereas their individual codons are typically recognized unambiguously as either sense or stops, solving the translation termination paradox.

In Search of tRNAs that Enable "Stop" Codon Translation

All model ciliates have "suppressor" tRNAs that are complementary to and permit translation of reassigned stop codons (Eisen et al., 2006; Hanyu et al., 1986; Kuchino et al., 1985). Although we found a comprehensive set of tRNAs in our *C. magnum* genome assemblies, including glutamine tRNAs capable of recognizing UAA and UAG codons (Figures 4A and 4B; Data S1G), we were unable to detect $tRNA^{Trp}$s with UCA anticodons. Given the high sequence coverage of the *C. magnum* macronuclear genome, it is unlikely that we missed $tRNA^{Trp}$(UCA)s. Ciliates possess both a micronuclear and a macronuclear genome, with the former predominantly unsequenced in our *C. magnum* assembly due to its comparatively low ploidy. It is also unlikely that $tRNA^{Trp}$(UCA)s have gone undetected because they are micronuclear genome-encoded: although these genomes are transcriptionally active during ciliate sexual development they are generally inactive during vegetative growth (Chen et al., 2014; Nowacki et al., 2009) when many transcripts with UGA tryptophan codons are expressed. To test if CCA → UCA anticodon editing produces a UGA-cognate $tRNA^{Trp}$, we sequenced RT-PCR products targeting nuclear genome-encoded $tRNA^{Trp}$s and examined tRNA reads from small RNA sequencing data, but found no signs of significant anticodon editing (see Supplemental Experimental Procedures).

All sequenced ciliate mitochondrial genomes encode a UGA-cognate $tRNA^{Trp}$(UCA) (Swart et al., 2013) and so does that of *C. magnum* (Figure S4A). Experiments in cell-free lysates show cytoplasmic ribosomes can use yeast mitochondrial

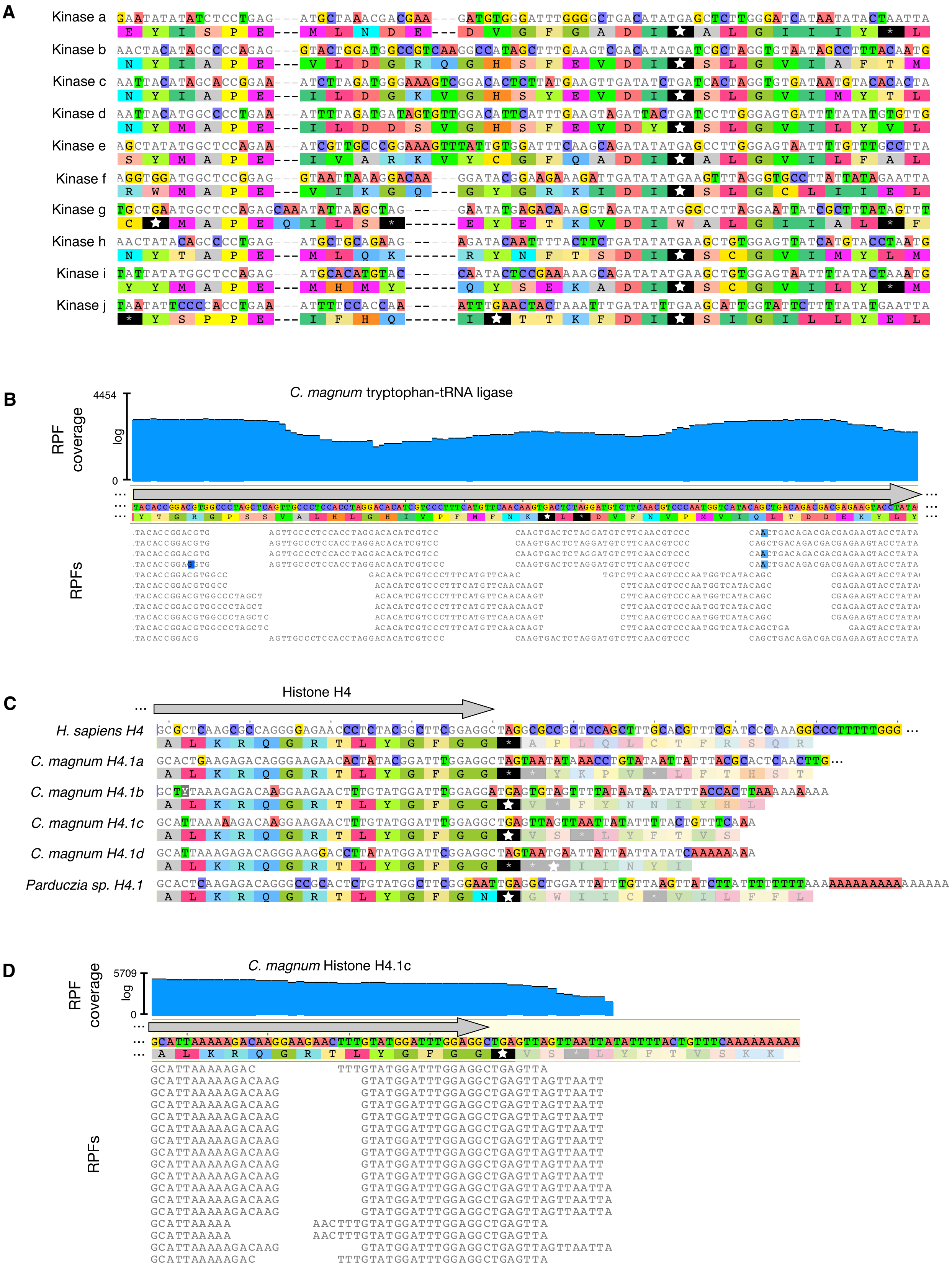

(legend on next page)

tRNA^{Trp}(UCA) to translate UGA codons (Tuite and McLaughlin, 1982). Thus, to determine whether *C. magnum*'s mitochondrial tRNA^{Trp}(UCA)s are used to translate its mRNA UGA codons, it will be necessary to show these tRNAs are accessible to cytoplasmic ribosomes in quantities adequate for translation.

In standard genetic code organisms, readthrough UGA stop codons are preferentially translated as tryptophan (e.g., for *Saccharomyces cerevisiae*: UGA: 86% W, 7% C, 7% R) (Roy et al., 2015) by near-cognate tRNA^{Trp}(CCA)s. Near-cognate pairing of tRNA^{Trp}(CCA) to UGA may also be substantially enhanced through particular mutations, e.g., in *Escherichia coli* a tRNA^{Trp}(CCA) D-stem point mutation leads to 30× more tryptophan translation at UGA stop codons than the wild-type tRNA (Hirsh, 1971; Hirsh and Gold, 1971). *C. magnum* has three types of tRNA^{Trp}(CCA) (Figures S4B and S4C), and it will be necessary to experimentally assess if any of these tRNAs permits efficient translation of its mRNA UGA codons.

"Stop" Codon Recognition Switches from Sense in Coding Sequences to Stop Near Transcript Ends

We assessed two hypotheses for how sense codons are distinguished from stop codons in ambiguous codes: (1) that there are sequence-specific features (motifs) allowing discriminating protein factors to bind nearby sense and stop codons, and (2) that proximity to transcript ends results in recognition of stops. We reject the hypothesis that specific sequences are necessary for stop/sense discrimination for the following reasons: (1) the base composition around sense "stop" codons is not constrained (Figure S5A), and (2) although the bases flanking *C. magnum* stop codons are weakly biased (Figure S5B), and such biases exist in other eukaryotes, where they are associated with enhanced termination efficiency (McCaughan et al., 1995), it is trivial to find sense "stop" codons with the preferred stop codon flanking Us, thus flanking bases cannot be sufficient to distinguish stop codons.

We next assessed if the proximity of the "stop" codon to transcript ends might determine sense/stop state. While analyzing ciliate 3′ UTRs we were struck by how short they are, with those of heterotrichs the shortest of all (median lengths, excluding the poly(A) tail and stop codon: 21–23 nt; Figure 5A). In the literature, we could find no eukaryotes with shorter 3′ UTRs. In comparison, yeast, metazoan, and plant 3′ UTRs typically have a >100 nt length mode and may be considerably longer (Aoki et al., 2010; Jan et al., 2011). Because poly(A) tails of certain *C. magnum* transcripts, especially those with UAA stop codons, start immediately after their stop codon (Figures 5B–5D) stops can be situated adjacent to poly(A)-binding proteins (PABPs) in vivo, and hence translation may be terminated with no additional information encoded by 3′ UTRs. Because the ribosome occupies 11 or 12 nucleotides downstream of *C. magnum* stop codons, even for those transcripts with 3′UTRs, there may be little room for ribosomes to maneuver passed stop codons without displacing PABPs. Given such short 3′ UTRs in ciliates, we therefore propose that nearby protein-bound poly(A) tails may contribute to discriminating stop from sense.

The very low readthrough levels detected in *C. magnum* by ribosome profiling imply that when "stop" codons are positioned close to transcript ends the probable outcome is termination. The few "stop" codons existing in the vicinity before stop codons (24–66 nt upstream; mean 50 nt upstream; 16 out of 1,672 transcripts) are efficiently translated and show no signs of appreciable premature translation termination (Figure S3I). Given the low tolerance of either readthrough or premature translation termination, the prediction is that when codons recognized inefficiently as either stop or sense arise in coding sequences, they are deleterious. Thus, in the hypothesis of discrimination of codons as stops close to transcript ends, if "stop" codons arise just upstream of the proper stops, where they might either be translated or result in premature termination, they will be counterselected and hence decrease in frequency. Consistent with this hypothesis, such a decrease in "stop" codon frequency exists in the upstream coding sequence vicinity of the stops in *C. magnum* (UAA, UAG, UGA) and *Parduczia* sp. (UGA) (Figures 6 and S6). Conversely, no codons other than "stop" codons become rare in coding sequences just before the actual stops (e.g., *C. magnum*; Figure S6). Furthermore, following cognate tRNA acquisition CAA and CAG frequencies are expected to remain higher near stops than distal coding sequence regions, since these codons may not freely mutate to UAA and UAG without causing premature translation termination (Figure 6D; unlike any other codons [Figure S6]; given the low UGA sense codon usage, only a small fraction of UGG codons has mutated to UGA, and UGG codon frequencies are not expected to be higher near stops).

DISCUSSION

Based on the observations of ribosome positioning and distribution of "stop" codons in transcripts, for translation in *C. magnum* and *Parduczia sp*. we propose a model where translation, rather than termination, is the default recognition mode for "stop" codons and where termination is due to the context-specific

Figure 2. "Stop" Codons in *C. magnum* and *Parduczia sp*.: Either Sense or Stop Codons

(A) *C. magnum* protein kinase alignment region highlighting putative sense "stop" codons. Standard genetic code stop codons are shown with stars, with larger stars for UGA. MMETSP0210 IDs: CAMNT_0008311047, CAMNT_0008316317, CAMNT_0008295895, CAMNT_0008281491, CAMNT_0008274923, CAMNT_0008274561, CAMNT_0008271577, CAMNT_0008291651, CAMNT_0008280967, CAMNT_0008289329.

(B) Ribosome-protected fragments (RPFs) mapped to a *C. magnum* tryptophan-tRNA ligase transcript (Data S1AC and S1AD). "RPF coverage" is calculated from all the bases of 25–32 nt RPFs.

(C) Histone H4 C-termini and stop codons (gray arrow, coding sequence) from *C. magnum*, *Parduczia sp*., and *Homo sapiens*. Poly(A) tails are visible at *C. magnum* and *Parduczia sp*. mRNA 3′ termini. Histone H4.1a– H4.1d: MMETSP0210 IDs: CAMNT_0008274265, CAMNT_0008297091, CAMNT_0008284521, and CAMNT_0008296393; *Parduczia sp*. histone H4 is MMETSP137 CAMNT_0047598059. *H. sapiens* histone H4 is GenBank: M16707.1. Judging from paired-end read mapping, the 3′ UTR of H4.1a is incorrectly fused to a downstream transcript.

(D) RPFs mapped to histone H4.1c (Data S1AE and S1AF).

See also Figure S2.

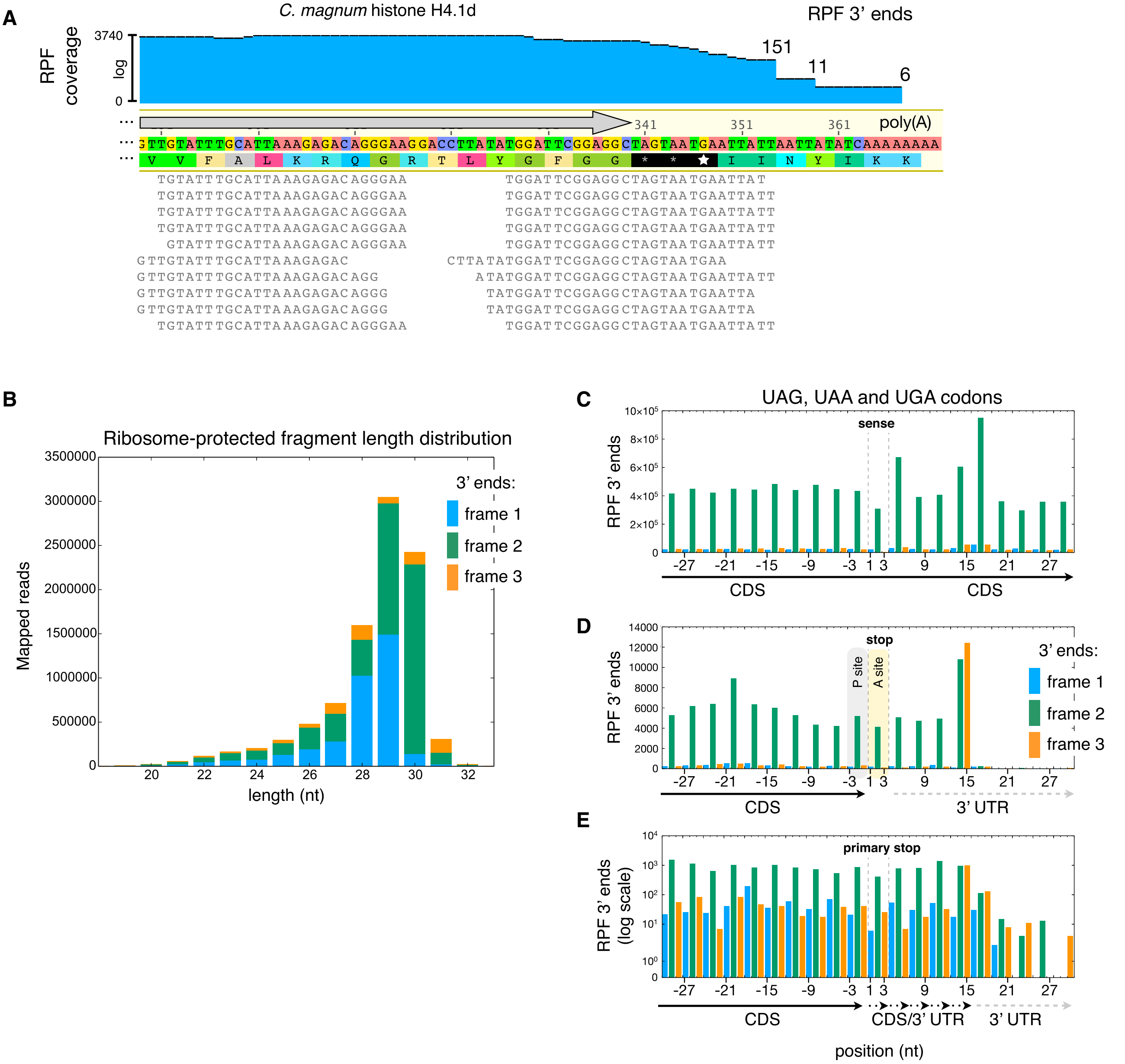

Figure 3. Ribosome Profiling Reveals Different Ribosome States at "Stop" Codons

(A) RPFs (25–32 nt) mapped to histone H4.1d (Data S1AG and S1AH). RPF 3′ termini counts are given at the sequence coverage steps: the first and second steps correspond to ribosomes whose P-sites are the first and second stop codons, respectively.

(B) RPF read length distribution and frame distribution. For the 3U TruSeq ribo profile nuclease digestion more mRNA reads were present due to lower rRNA degradation, and most 30-nt RPFs have their 3′ ends in frame 3 (compare to Figures S3A and S3B).

(C and D) Distribution of 30 nt RPF 3′ ends around sense (C) and stop (D) UAG, UGA, and UAA codons (positions 1–3, indicated by dashed vertical lines) in Trinity assembled transcripts. CDS, coding sequence; UTR, untranslated region. Putative ribosomal P- and A-site locations of translation terminating RPFs situated at stop codons, based on that predicted for other eukaryotic ribosomes (Chung et al., 2015). Figures S3C–S3H show the distribution of RPF 3′ ends around individual "stop" codons. Though the termination signal is most pronounced for 30-nt RPFs, it is also exhibited by other RPFs (Figure S3J).

(E) Distribution of 30-nt RPFs for transcripts with detected readthrough (≥ 13 nt downstream of the primary stop codon); additional stop codons are located downstream of the primary one, hence the region downstream of the primary stop may be either coding or untranslated.

See also Figure S3.

override provided by transcript ends (Figure 7). Thus, at sense "stop" codons, tRNAs outcompete eRF1, and at proper stop codons, eRF1 outcompetes tRNAs. The converse model (default termination; context-specific translation), is not consistent with our results, and given preexisting surrounding coding sequence constraints, widespread context-specific translation signals

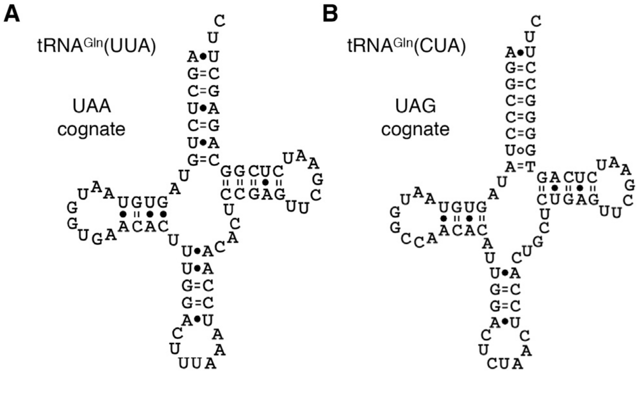

Figure 4. Predicted UAA- and UAG-Cognate *C. magnum* tRNAs

(A and B) UAA- and UAG-cognate glutamine tRNA secondary structures. Bonds shown are predicted by the RNAfold web server (Lorenz et al., 2011) (default parameters).

See also Figure S4.

necessary to translate all the "stop" codons are exceedingly unlikely to arise.

Given the existence of transcripts without 3′ UTRs, we deduce these regions are not essential for translation termination, and we propose that the close proximity of a poly(A) tail and poly(A)-interacting proteins, in particular PABPs, alone may be necessary to trigger termination. Three prior observations favor this hypothesis: (1) PABP overexpression enhances translation termination when it is weak, implying that PABPs may be involved in translation termination (Cosson et al., 2002), (2) tethering of a PABP 37–73 nt downstream of a premature stop codon substantially decreases NMD and results in recruitment of the translation termination factor eRF3, suggesting that PABP is involved in discriminating stops from premature stops (Amrani et al., 2004); and (3) PABPs bind to AU-rich RNA including 3′ UTRs (Baejen et al., 2014; Kini et al., 2016; Sladic et al., 2004).

Reassigned "stop" codons in *C. magnum* and *Parduczia sp.* differ from conventional readthrough stops in standard genetic code organisms because they are efficiently translated and distributed throughout coding sequences, whereas conventional readthrough stops are the major termination signals whose disregard gives rise to modest levels of short protein extensions (Dunn et al., 2013; Jungreis et al., 2011). From their distribution throughout coding sequences, it is evident that most reassigned codons in ciliates arose from substitutions of codons that were already normally translated, rather than from readthrough stop codons. Upon acquisition of a stop cognate tRNA, a shift in balance from translation termination to readthrough at stop codons is expected. Normally this acquisition would immediately be deleterious, due to the creation of aberrant C-terminal peptide signals or the triggering of non-stop mRNA decay (Frischmeyer et al., 2002) upon translation into mRNA poly(A) tails. By enforcing proper translation termination close to transcript ends, ciliates with ambiguous genetic codes provide a way of getting around these problems.

Given that we detected no new genetic codes in 265 diverse non-ciliate eukaryotic species from MMETSP, the abundance of alternative genetic codes within ciliates is all the more striking. Two hypotheses for the origin of genetic codes in ciliates are that they were enabled by codon capture or eRF1 mutations. Under the "codon capture" hypothesis (Osawa and Jukes, 1989) when a codon disappears in a genome due to strong mutational biases it may then be reassigned when a suitable cognate tRNA arises (via tRNA duplication and anticodon mutation) and the codon subsequently reappears. To date, all sequenced ciliate genomes are AT rich (Aeschlimann et al., 2014; Aury et al., 2006; Coyne et al., 2011; Eisen et al., 2006; Swart et al., 2013; Wang et al., 2016). Reflecting their A/T mutational biases, among eukaryotes with the highest UAA stop codon usage are standard genetic code ciliates (Figures S7B–S7D; Data S1V). This suggests that the diversification of genetic codes from the standard one could have followed UAG and UGA stop codon depletion in ancestral ciliates with AT rich genomes. While codon capture is a reasonable explanation for the evolution of the *Blepharisma* genetic code (UAA stop codon usage 91%), it does not readily explain the origin of other ciliate genetic codes. For example, in *Euplotes sp.*, according to tRNA anticodon-codon wobble rules, UGG codons are expected to be misread as cysteine following the origin of a $tRNA^{Cys}$(UCA).

Even when relaxing the stop codon disappearance criterion (via genetic code ambiguity tolerance), codon capture cannot easily explain the general UAG and UAA reassignment trends seen in Figure 1A. In all ciliates with reassigned UAG and UAA codons and complete macronuclear genomes, both tRNAs with anticodon complements of these codons are present (Aeschlimann et al., 2014; Aury et al., 2006; Coyne et al., 2011; Eisen et al., 2006; Swart et al., 2013). In the event that the first acquisition during codon reassignment was a tRNA(UUA), by the codon-anticodon wobble rules UAA and UAG would both be translated; however, as this requires prior UAA stop codon disappearance, it is contrary to the ciliate mutational tendencies. If codon reassignment were to occur after a tRNA(CUA) acquisition, only UAG codons would be translated, and under the codon capture hypothesis, genetic codes with UAG reassignment alone should be common; however, this is not observed. Therefore, codon capture alone cannot explain the diversity of genetic codes in ciliates.

As eRF1 recognizes stop codons, this protein could be a determinant of genetic code reassignments in ciliates. Previously it was hypothesized that particular eRF1 amino acid substitutions are associated with each variant genetic code (Lozupone et al., 2001). The additional ciliate genetic codes and eRF1 diversity present in ciliates and other eukaryotes present multiple contradictions to the reported concordances between eRF1 amino acid substitutions and variant genetic codes (Lozupone et al., 2001) (Figure S7A). Because no obvious associations between single eRF1 substitutions and variant genetic codes are evident, any possible associations between genetic codes and eRF1 changes must be more complex than individual amino acid changes. The existence of the ambiguous ciliate genetic codes is also a challenge to explain by this hypothesis.

Because ciliate genetic code diversity does not seem to be adequately explained by codon capture or eRF1 changes, we instead propose that it is due to past genetic code ambiguity tolerance and resolution, as exemplified by *C. magnum* and *Parduczia sp.* Conversely, the inability to resolve ambiguity favors the "frozen" state of the genetic code in other eukaryotes.

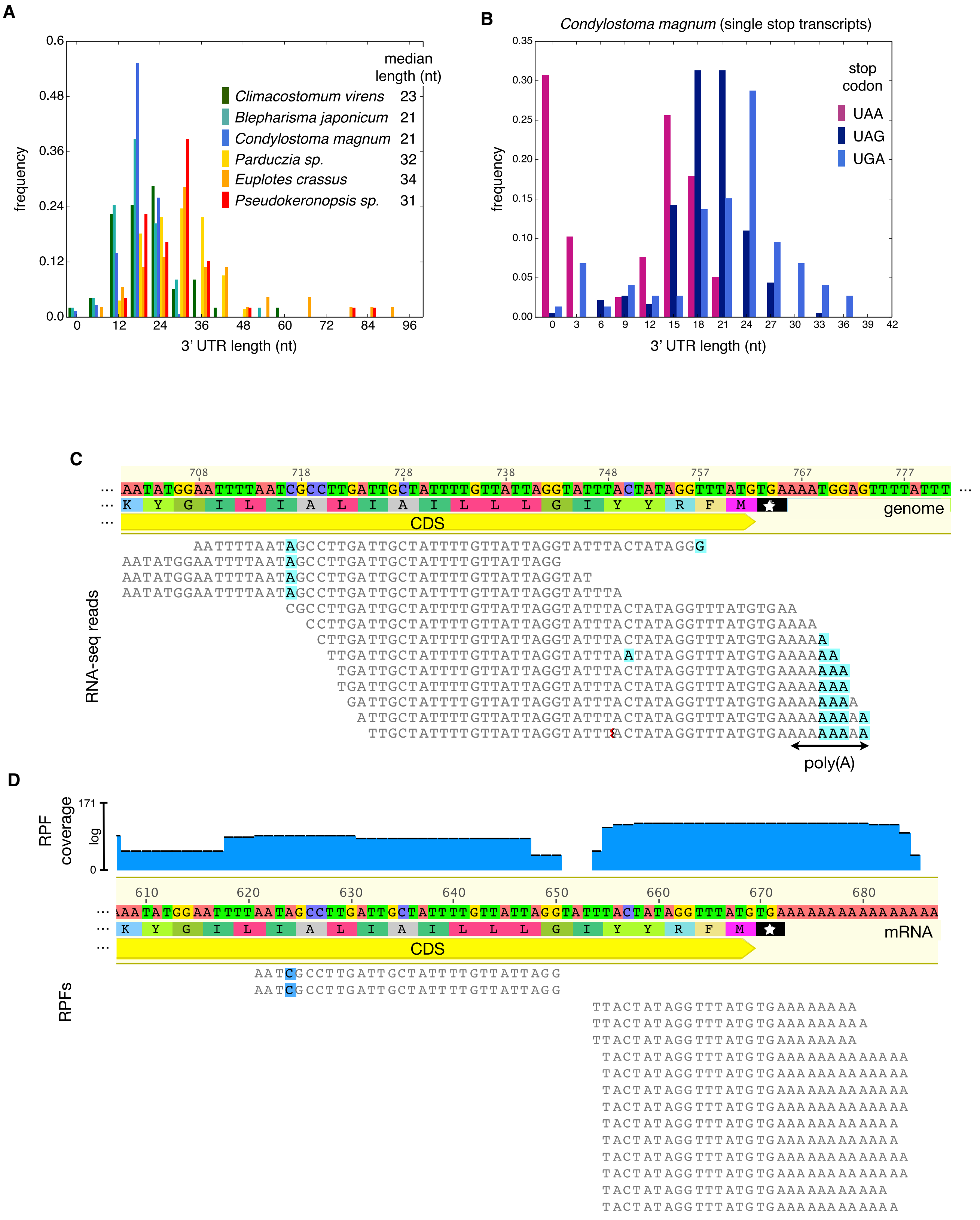

(legend on next page)

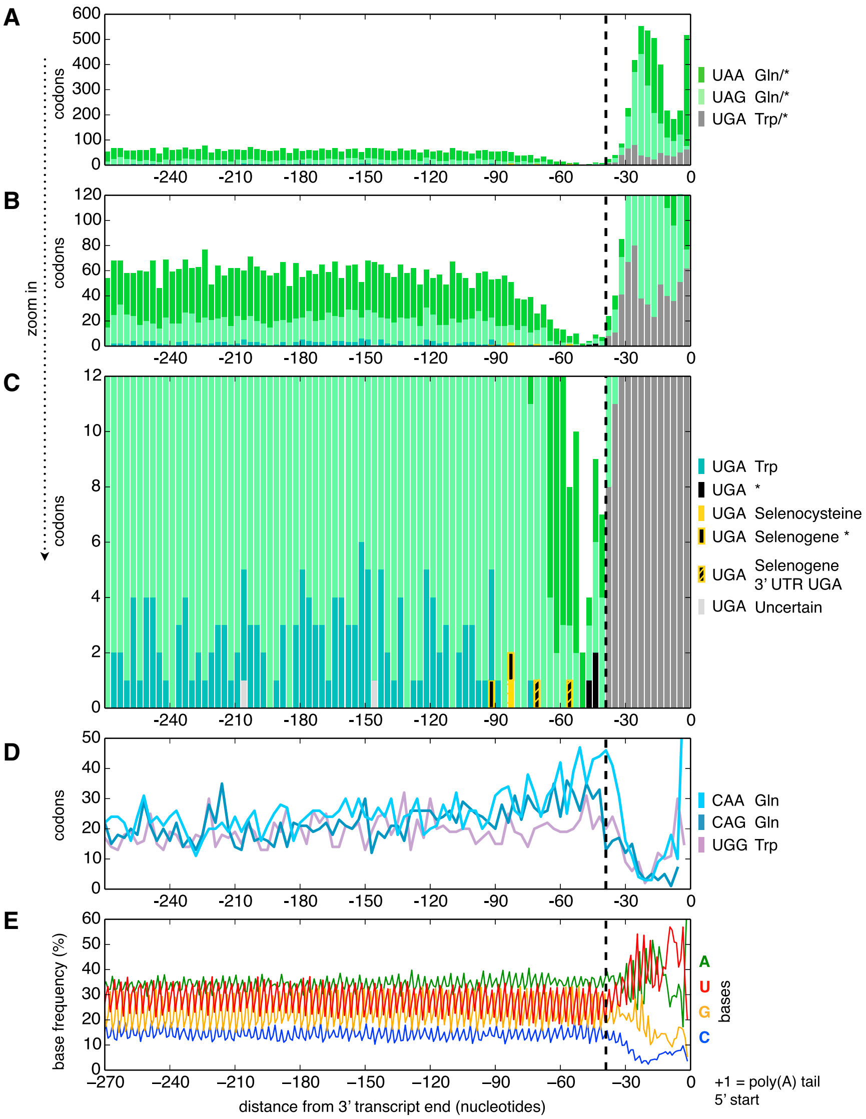

Figure 6. Terminal "Stop" Codon Decline Close to *C. magnum* Stops

Stacked bar graphs of "stop" codon counts are for the transcript regions upstream of poly(A) tails (position 0). Transcript ends include 0, 1, or 2 nucleotides of the poly(A) tail to complete the final "codon." 3′ UTRs occur in the region to the right of the right-most dashed vertical line. Codons counted are those in the 1672 poly(A)-tailed single gene, single isoform Trinity assembled transcripts.

(A–C) The top three subgraphs are drawn in decreasing order of ordinate limits. Vertical line at −39 nt indicates approximately where most downstream "stops" are either stop codons or "codons" in 3′ UTRs. Codons whose sense/stop states have not been determined are indicated by "amino acid/*." Transcripts with UGA codons upstream of −39 nt were visually classified based on BLASTX searches. Upstream of −39 nt, UGA codons predominantly code for tryptophan; downstream of −39 nt, UGA codons are predominantly stops or codons in 3′ UTRs downstream of primary stops (both indicated by gray bars). In the genetic codes of *C. magnum* and *Parduczia sp*. UGA is a codon triality (codon duality is reviewed in Atkins and Baranov, 2007), because in addition to being interpreted as a tryptophan codon and a stop codon, it also serves as a selenocysteine codon in the context of SECIS elements. Pale gray bars correspond to a transcript with an uncertain C-terminal, as judged by BLAST.

(D) Standard glutamine and tryptophan sense codon counts.

(E) Base frequencies are stable in the region of "stop" codon decline (∼−90 to −42 bases upstream of poly-As).

See also Figures S5 and S6.

The codons in *C. magnum* and *Parduczia sp.* that are recognized either by tRNAs or eRF1 represent precisely the type of intermediate states with multiple meanings originally proposed to occur in the hypothesis of genetic code evolution through ambiguous translational intermediates (Schultz and Yarus, 1994). We furthermore propose that the evolution of very short, AU-rich 3′ UTRs and termination facilitated by poly(A) proximity have enabled codon reassignment, as translational ambiguity due to the acquisition of stop cognate tRNAs could be suppressed at stops.

In light of the ambiguous genetic codes presented here, it is worth reconsidering the idea that the standard genetic code is "one in a million" and is optimized to minimize the effects of errors arising from mutations (Freeland and Hurst, 1998) (although contested [Koonin and Novozhilov, 2009]). Naturally, organisms with only one or two stop codons are more robust to sense premature stop codon mutations than those with the standard genetic code. Given that, other than in the vicinity of transcript ends, "stop" codons are translated by default, the genetic codes of *C. magnum* and *Parduczia sp.* may confer very high resistance

Figure 5. Extremely Short and Nonexistent 3′ UTRs in Heterotrichs

(A) Ciliate 3′ UTR length distributions (lengths exclude the stop codon and poly(A) tail) for representatives of the ciliate genetic codes in Figure 1.

(B) Length distribution of *C. magnum* 3′ UTRs. Lengths are from the putative primary stop in the 60 nt window upstream of poly(A) sites and exclude the stop and poly(A) tail lengths.

(C) A 3′ UTR-less gene (synaptobrevin homolog). Poly(A) tail-ending reads mapped to the genomic region encoding this gene are shown, and no other reads extend beyond the poly(A) addition site. CDS, coding sequence (Data S1AI and S1AJ).

(D) RPFs mapped to a transcript of the gene in (C) (Data S1AK and S1AL).

See also Figure S6.

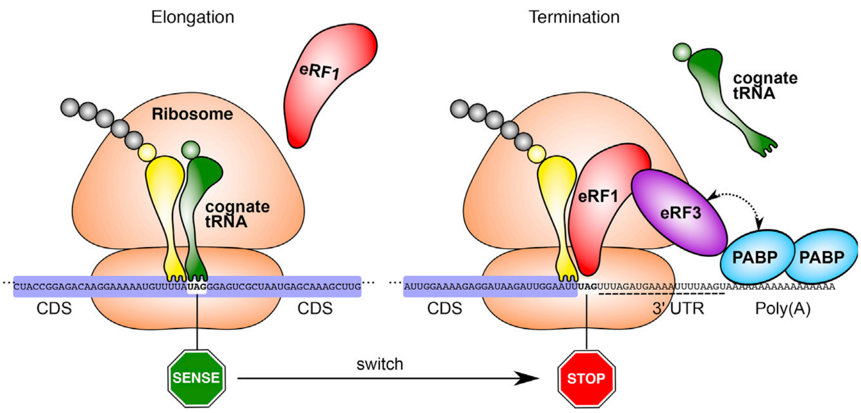

Figure 7. Model for Distinguishing Stops from Sense "Stops"

Representative regions from the same transcript (MMETSP0210: CAMNT_0008285195), with translation through a UAG sense codon and termination at a UAG stop codon (codon state verified by ribo-seq). CDS, coding sequence; 3′ UTR, 3′ UTR; eRF1, eukaryotic release factor 1; eRF3, eukaryotic release factor 3; PABP, poly(A)-binding protein; standard amino acids are indicated by circles. Putative interaction between eRF3 and PABPs, as inferred from experimental evidence in yeast (Cosson et al., 2002), is indicated by a dotted bidirectional arrow. Ribosome position and the protected mRNA span are illustrated as inferred from *C. magnum* RPFs and from estimates of other eukaryotic ribosomes (Chung et al., 2015).

to substitutions that would cause premature translation termination in the standard genetic code. A potential drawback of such robustness is that large insertions at 3′ transcript ends may expose stops that were previously translated. However, large insertions likely occur much less often than substitutions, and the strong purifying selection governing non-protein-coding regions in the heterotrich and karyorelict genomes will inhibit progressive transcript end lengthening.

In summary, we propose that ambiguous ciliate genetic codes are resolved by context-dependent translation termination, and the reason why ciliates possess such diverse genetic codes is that their ancestors had the ability to thrive for extended periods with ambiguous genetic codes, as epitomized by *C. magnum*. Together with the other variant genetic codes, these codes show that the standard nuclear genetic code is not necessarily an evolutionary dead end and that genetic codes can occasionally be observed in a state of flux. As highlighted here, the ambiguous genetic codes of *C. magnum* and *Parduczia sp.* also have ramifications for our understanding of the suppression of translational readthrough, as well as how nonsense-mediated decay (NMD) and selenocysteine translation operate (conserved proteins from both of these pathways are present in ciliates with ambiguous genetic codes; see e.g., Figure S2E). To facilitate future investigations concerning how sense is distinguished from stop and related questions about codon disambiguation, we have made a draft *C. magnum* macronuclear genome available under the accession number European Nucleotide Archive: GCA_001499635.1.

EXPERIMENTAL PROCEDURES

See the Supplemental Experimental Procedures for additional detailed protocols.

Transcriptomes Analyzed

Transcriptomes for *C. magnum* (MMETSP0210), *Parduczia sp.* (MMETSP1317), and other eukaryotes assembled as part of MMETSP (Gentekaki et al., 2014; Keeling et al., 2014)) were used to identify genetic codes and analyze stop codon usage. We also predicted genetic codes after de novo assembling the transcriptomes of two peritrichous ciliates: *Campanella umbellaria* and *Carchesium polypinum* (NCBI short read archive: SRR1768423 and SRR1768437, respectively; data from a recent phylogenomic study) (Feng et al., 2015) with Trinity (Grabherr et al., 2011) (default parameters, version: trinityrnaseq_r20140717).

Prediction of Alternative Stop Codon Reassignments

To predict codon reassignments, we simplified and refined the key steps of a method developed for such prediction (Dutilh et al., 2011), which identifies codons aligned to conserved amino acids in hidden Markov models inferred from multiple sequence alignments. Dutilh et al. (2011) may be consulted for a graphical outline and more details of the method. This method builds upon and advances the classical method of inspecting conserved positions in multiple sequence alignments of homologous protein sequences to infer codon reassignments. First, we generated a database of peptide sequences by translating nucleotide sequences in all six frames with the standard genetic code, recording standard stop codons as "X" (any amino acid). Next, we used HMMER 3.1b (http://hmmer.org) to search and align the hidden Markov models from the Pfam-A protein domain database (release 27) (Finn et al., 2014) against the translated sequences. Using a custom Python script, the alignment outputs were filtered at a conditional e-value threshold <1e-10. We then simultaneously scanned through the Pfam consensus, aligned database match and its underlying coding sequence, recording the codon and consensus amino acid for well-conserved amino acids at $\geq$50% frequency in columns of the multiple sequence alignment used to build the Pfam model. From the resultant counts of aligned amino acid/codon pairs ($m_{i,j}$; i = 1..64 codons, j = 1..20 amino acids) a 20 amino acid by 64 codon matrix, *M*, was created, with each entry scaled by the sum of the counts for each amino acid (i.e., $M = m_{i,j}/\sum_i m_{i,j}$). This matrix was used to generate a sequence logo with WebLogo 3.3 (Crooks et al., 2004) (command line switches: "–scale-width no -c chemistry -U probability -A protein"). Note that the lower frequency amino acids shown in the genetic code logos generated by this procedure typically reflect the underlying codon mutational space, but may also be subject to noise, and the focus for codon reassignment prediction should be on the highest frequency amino acid. Genetic code sequence logos for all MMETSP transcriptomes are provided as Data S1A (ciliates) and Data S1B (nonciliates). See Table S1 for a summary of the ciliate genetic code predictions. An explanation of stop codon identification is provided in the Supplemental Experimental Procedures.

Ribosome Profiling

Illumina's TruSeq Ribo Profile (Mammalian) kit was used for ribosome profiling. A total of 32,000 *C. magnum* cells (strain COL2) were isolated, gently pelleted at 280 × *g* for 2 min in 100 ml pear-shaped centrifuge tubes, then washed in clean saline solution and centrifuged again at 280 × *g* for 2 min to remove excess algae. The cleaned *C. magnum* cell pellet was incubated in saline solution with 0.1 mg/ml cycloheximide for 1 min. Cells were rinsed with 10 ml PBS, 0.1 mg/ml cycloheximide, pelleted at 280 × *g*, and excess liquid was removed with a micropipette. Pelleted cells were lysed in TruSeq Ribo Profile lysis buffer using a syringe with a 21G needle. The TruSeq Ribo Profile protocol was followed for the remaining ribosome profiling steps. Three concentrations of TruSeq Ribo Profile Nuclease (3 U, 10 U, and 30 U) were used to generate ribosome-protected fragments (RPFs), which were purified with MicroSpin S-400 columns. Ribo-Zero Gold Yeast rRNA depletion was performed on purified RPFs. DNA libraries isolated from 15 (10 U) or 17 (3 U, 10 U) cycle PCRs

were multiplexed and sequenced on one lane of a HiSeq 2500 sequencer by Fasteris SA (Switzerland). Ribosome profiling data are available from the European Nucleotide Archive: ERS1066482–ERS1066484. After adaptor trimming, reads were mapped to 1,672 poly(A)-tailed, translation frame inferred Trinity assembled transcripts (see the Supplemental Experimental Procedures) with STAR (parameters:"–alignIntronMin 12 –alignIntronMax 25"). Reads with 0 or 1 mismatches to the transcripts were used in ribo-seq analyses.

ACCESSION NUMBERS

The accession number for the draft of the *C. magnum* macronuclear genome reported in this paper is European Nucleotide Archive: GCA_001499635.1.

SUPPLEMENTAL INFORMATION

Supplemental Information includes Supplemental Experimental Procedures, seven figures, one table, and supplemental data and can be found with this article online at http://dx.doi.org/10.1016/j.cell.2016.06.020.

AUTHOR CONTRIBUTIONS

E.C.S. performed the computational analyses and assisted in laboratory experiments. V.S. cultured *C. magnum*, isolated nucleic acids and proteins, and performed laboratory experiments searching for tRNAs. E.C.S. and V.S. performed ribosome profiling. M.N. supervised the project. E.C.S. drafted the manuscript with input from V.S., G.P., and M.N.

ACKNOWLEDGMENTS

We thank Letizia Modeo for collecting *C. magnum*, Vittorio Boscaro for the original *C. magnum* RNA isolation, Sophie Braga-Lagache and Manfred Heller from the Mass Spectrometry and Proteomics Laboratory at the Children's University Hospital in Bern for mass spectrometry support, Deis Haxholli for initial genetic code inspections and the M.N. lab members for support and discussion. This research was supported by grants from the European Research Council (ERC) (EPIGENOME) and National Center of Competence in Research (NCCR) RNA and Disease to M.N., and the European COST Action BM1102. Cluster computing was performed at the Vital-IT Center for High-Performance Computing (http://www.vital-it.ch) of the Swiss Institute of Bioinformatics.

Received: August 11, 2015
Revised: April 19, 2016
Accepted: June 6, 2016
Published: July 14, 2016

REFERENCES

Aeschlimann, S.H., Jönsson, F., Postberg, J., Stover, N.A., Petera, R.L., Lipps, H.J., Nowacki, M., and Swart, E.C. (2014). The draft assembly of the radically organized Stylonychia lemnae macronuclear genome. Genome Biol. Evol. *6*, 1707–1723.

Amrani, N., Ganesan, R., Kervestin, S., Mangus, D.A., Ghosh, S., and Jacobson, A. (2004). A faux 3′-UTR promotes aberrant termination and triggers nonsense-mediated mRNA decay. Nature *432*, 112–118.

Aoki, K., Yano, K., Suzuki, A., Kawamura, S., Sakurai, N., Suda, K., Kurabayashi, A., Suzuki, T., Tsugane, T., Watanabe, M., et al. (2010). Large-scale analysis of full-length cDNAs from the tomato (Solanum lycopersicum) cultivar Micro-Tom, a reference system for the Solanaceae genomics. BMC Genomics *11*, 210.

Atkins, J.F., and Baranov, P.V. (2007). Translation: duality in the genetic code. Nature *448*, 1004–1005.

Aury, J.M., Jaillon, O., Duret, L., Noel, B., Jubin, C., Porcel, B.M., Ségurens, B., Daubin, V., Anthouard, V., Aiach, N., et al. (2006). Global trends of whole-genome duplications revealed by the ciliate Paramecium tetraurelia. Nature *444*, 171–178.

Baejen, C., Torkler, P., Gressel, S., Essig, K., Söding, J., and Cramer, P. (2014). Transcriptome maps of mRNP biogenesis factors define pre-mRNA recognition. Mol. Cell *55*, 745–757.

Blanchet, S., Cornu, D., Argentini, M., and Namy, O. (2014). New insights into the incorporation of natural suppressor tRNAs at stop codons in Saccharomyces cerevisiae. Nucleic Acids Res. *42*, 10061–10072.

Caron, F., and Meyer, E. (1985). Does Paramecium primaurelia use a different genetic code in its macronucleus? Nature *314*, 185–188.

Chen, X., Bracht, J.R., Goldman, A.D., Dolzhenko, E., Clay, D.M., Swart, E.C., Perlman, D.H., Doak, T.G., Stuart, A., Amemiya, C.T., et al. (2014). The architecture of a scrambled genome reveals massive levels of genomic rearrangement during development. Cell *158*, 1187–1198.

Chung, B.Y., Hardcastle, T.J., Jones, J.D., Irigoyen, N., Firth, A.E., Baulcombe, D.C., and Brierley, I. (2015). The use of duplex-specific nuclease in ribosome profiling and a user-friendly software package for Ribo-seq data analysis. RNA *21*, 1731–1745.

Cosson, B., Couturier, A., Chabelskaya, S., Kiktev, D., Inge-Vechtomov, S., Philippe, M., and Zhouravleva, G. (2002). Poly(A)-binding protein acts in translation termination via eukaryotic release factor 3 interaction and does not influence [PSI(+)] propagation. Mol. Cell. Biol. *22*, 3301–3315.

Coyne, R.S., Hannick, L., Shanmugam, D., Hostetler, J.B., Brami, D., Joardar, V.S., Johnson, J., Radune, D., Singh, I., Badger, J.H., et al. (2011). Comparative genomics of the pathogenic ciliate Ichthyophthirius multifiliis, its free-living relatives and a host species provide insights into adoption of a parasitic lifestyle and prospects for disease control. Genome Biol. *12*, R100.

Crooks, G.E., Hon, G., Chandonia, J.M., and Brenner, S.E. (2004). WebLogo: a sequence logo generator. Genome Res. *14*, 1188–1190.

Dunn, J.G., Foo, C.K., Belletier, N.G., Gavis, E.R., and Weissman, J.S. (2013). Ribosome profiling reveals pervasive and regulated stop codon readthrough in Drosophila melanogaster. eLife *2*, e01179.

Dutilh, B.E., Jurgelenaite, R., Szklarczyk, R., van Hijum, S.A., Harhangi, H.R., Schmid, M., de Wild, B., Françoijs, K.J., Stunnenberg, H.G., Strous, M., et al. (2011). FACIL: Fast and Accurate Genetic Code Inference and Logo. Bioinformatics *27*, 1929–1933.

Edgcomb, V.P., Leadbetter, E.R., Bourland, W., Beaudoin, D., and Bernhard, J.M. (2011). Structured multiple endosymbiosis of bacteria and archaea in a ciliate from marine sulfidic sediments: a survival mechanism in low oxygen, sulfidic sediments? Front Microbiol *2*, 55.

Eisen, J.A., Coyne, R.S., Wu, M., Wu, D., Thiagarajan, M., Wortman, J.R., Badger, J.H., Ren, Q., Amedeo, P., Jones, K.M., et al. (2006). Macronuclear genome sequence of the ciliate Tetrahymena thermophila, a model eukaryote. PLoS Biol. *4*, e286.

Eliseev, B., Kryuchkova, P., Alkalaeva, E., and Frolova, L. (2011). A single amino acid change of translation termination factor eRF1 switches between bipotent and omnipotent stop-codon specificity. Nucleic Acids Res. *39*, 599–608.

Feng, J.M., Jiang, C.Q., Warren, A., Tian, M., Cheng, J., Liu, G.L., Xiong, J., and Miao, W. (2015). Phylogenomic analyses reveal subclass Scuticociliatia as the sister group of subclass Hymenostomatia within class Oligohymenophorea. Mol. Phylogenet. Evol. *90*, 104–111.

Finn, R.D., Bateman, A., Clements, J., Coggill, P., Eberhardt, R.Y., Eddy, S.R., Heger, A., Hetherington, K., Holm, L., Mistry, J., et al. (2014). Pfam: the protein families database. Nucleic Acids Res. *42*, D222–D230.

Freeland, S.J., and Hurst, L.D. (1998). The genetic code is one in a million. J. Mol. Evol. *47*, 238–248.

Frischmeyer, P.A., van Hoof, A., O'Donnell, K., Guerrerio, A.L., Parker, R., and Dietz, H.C. (2002). An mRNA surveillance mechanism that eliminates transcripts lacking termination codons. Science *295*, 2258–2261.

Gentekaki, E., Kolisko, M., Boscaro, V., Bright, K.J., Dini, F., Di Giuseppe, G., Gong, Y., Miceli, C., Modeo, L., Molestina, R.E., et al. (2014). Large-scale phylogenomic analysis reveals the phylogenetic position of the problematic taxon Protocruzia and unravels the deep phylogenetic affinities of the ciliate lineages. Mol. Phylogenet. Evol. *78*, 36–42.

Gomes, A.C., Miranda, I., Silva, R.M., Moura, G.R., Thomas, B., Akoulitchev, A., and Santos, M.A. (2007). A genetic code alteration generates a proteome of high diversity in the human pathogen Candida albicans. Genome Biol. *8*, R206.

Grabherr, M.G., Haas, B.J., Yassour, M., Levin, J.Z., Thompson, D.A., Amit, I., Adiconis, X., Fan, L., Raychowdhury, R., Zeng, Q., et al. (2011). Full-length transcriptome assembly from RNA-Seq data without a reference genome. Nat. Biotechnol. *29*, 644–652.

Hanyu, N., Kuchino, Y., Nishimura, S., and Beier, H. (1986). Dramatic events in ciliate evolution: alteration of UAA and UAG termination codons to glutamine codons due to anticodon mutations in two Tetrahymena tRNAs. EMBO J. *5*, 1307–1311.

Harrell, L., Melcher, U., and Atkins, J.F. (2002). Predominance of six different hexanucleotide recoding signals 3′ of read-through stop codons. Nucleic Acids Res. *30*, 2011–2017.

Helftenbein, E. (1985). Nucleotide sequence of a macronuclear DNA molecule coding for alpha-tubulin from the ciliate Stylonychia lemnae. Special codon usage: TAA is not a translation termination codon. Nucleic Acids Res. *13*, 415–433.

Hirsh, D. (1971). Tryptophan transfer RNA as the UGA suppressor. J. Mol. Biol. *58*, 439–458.

Hirsh, D., and Gold, L. (1971). Translation of the UGA triplet in vitro by tryptophan transfer RNA's. J. Mol. Biol. *58*, 459–468.

Horowitz, S., and Gorovsky, M.A. (1985). An unusual genetic code in nuclear genes of Tetrahymena. Proc. Natl. Acad. Sci. USA *82*, 2452–2455.

Jan, C.H., Friedman, R.C., Ruby, J.G., and Bartel, D.P. (2011). Formation, regulation and evolution of Caenorhabditis elegans 3'UTRs. Nature *469*, 97–101.

Johnson, M.D., Tengs, T., Oldach, D.W., Delwiche, C.F., and Stoecker, D.K. (2004). Highly divergent SSU rRNA genes found in the marine ciliates Myrionecta rubra and Mesodinium pulex. Protist *155*, 347–359.

Jungreis, I., Lin, M.F., Spokony, R., Chan, C.S., Negre, N., Victorsen, A., White, K.P., and Kellis, M. (2011). Evidence of abundant stop codon readthrough in Drosophila and other metazoa. Genome Res. *21*, 2096–2113.

Keeling, P.J., and Doolittle, W.F. (1996). A non-canonical genetic code in an early diverging eukaryotic lineage. EMBO J. *15*, 2285–2290.

Keeling, P.J., Burki, F., Wilcox, H.M., Allam, B., Allen, E.E., Amaral-Zettler, L.A., Armbrust, E.V., Archibald, J.M., Bharti, A.K., Bell, C.J., et al. (2014). The Marine Microbial Eukaryote Transcriptome Sequencing Project (MMETSP): illuminating the functional diversity of eukaryotic life in the oceans through transcriptome sequencing. PLoS Biol. *12*, e1001889.

Kini, H.K., Silverman, I.M., Ji, X., Gregory, B.D., and Liebhaber, S.A. (2016). Cytoplasmic poly(A) binding protein-1 binds to genomically encoded sequences within mammalian mRNAs. RNA *22*, 61–74.

Knight, R.D., Freeland, S.J., and Landweber, L.F. (2001). Rewiring the keyboard: evolvability of the genetic code. Nat. Rev. Genet. *2*, 49–58.

Koonin, E.V., and Novozhilov, A.S. (2009). Origin and evolution of the genetic code: the universal enigma. IUBMB Life *61*, 99–111.

Kuchino, Y., Hanyu, N., Tashiro, F., and Nishimura, S. (1985). Tetrahymena thermophila glutamine tRNA and its gene that corresponds to UAA termination codon. Proc. Natl. Acad. Sci. USA *82*, 4758–4762.

Lekomtsev, S., Kolosov, P., Bidou, L., Frolova, L., Rousset, J.P., and Kisselev, L. (2007). Different modes of stop codon restriction by the Stylonychia and Paramecium eRF1 translation termination factors. Proc. Natl. Acad. Sci. USA *104*, 10824–10829.

Lemke, E.A. (2014). The exploding genetic code. ChemBioChem *15*, 1691–1694.

Lorenz, R., Bernhart, S.H., Höner Zu Siederdissen, C., Tafer, H., Flamm, C., Stadler, P.F., and Hofacker, I.L. (2011). ViennaRNA Package 2.0. Algorithms Mol. Biol. *6*, 26.

Lozupone, C.A., Knight, R.D., and Landweber, L.F. (2001). The molecular basis of nuclear genetic code change in ciliates. Curr. Biol. *11*, 65–74.

McCaughan, K.K., Brown, C.M., Dalphin, M.E., Berry, M.J., and Tate, W.P. (1995). Translational termination efficiency in mammals is influenced by the base following the stop codon. Proc. Natl. Acad. Sci. USA *92*, 5431–5435.

Mühlhausen, S., Findeisen, P., Plessmann, U., Urlaub, H., and Kollmar, M. (2016). A novel nuclear genetic code alteration in yeasts and the evolution of codon reassignment in eukaryotes. Genome Res. Published online May 6, 2016. http://dx.doi.org/10.1101/gr.200931.115.

Nasim, M.T., Jaenecke, S., Belduz, A., Kollmus, H., Flohé, L., and McCarthy, J.E. (2000). Eukaryotic selenocysteine incorporation follows a nonprocessive mechanism that competes with translational termination. J. Biol. Chem. *275*, 14846–14852.

Nowacki, M., Higgins, B.P., Maquilan, G.M., Swart, E.C., Doak, T.G., and Landweber, L.F. (2009). A functional role for transposases in a large eukaryotic genome. Science *324*, 935–938.

Osawa, S., and Jukes, T.H. (1989). Codon reassignment (codon capture) in evolution. J. Mol. Evol. *28*, 271–278.

Preer, J.R., Jr., Preer, L.B., Rudman, B.M., and Barnett, A.J. (1985). Deviation from the universal code shown by the gene for surface protein 51A in Paramecium. Nature *314*, 188–190.

Roy, B., Leszyk, J.D., Mangus, D.A., and Jacobson, A. (2015). Nonsense suppression by near-cognate tRNAs employs alternative base pairing at codon positions 1 and 3. Proc. Natl. Acad. Sci. USA *112*, 3038–3043.

Salas-Marco, J., Fan-Minogue, H., Kallmeyer, A.K., Klobutcher, L.A., Farabaugh, P.J., and Bedwell, D.M. (2006). Distinct paths to stop codon reassignment by the variant-code organisms Tetrahymena and Euplotes. Mol. Cell. Biol. *26*, 438–447.

Sánchez-Silva, R., Villalobo, E., Morin, L., and Torres, A. (2003). A new noncanonical nuclear genetic code: translation of UAA into glutamate. Curr. Biol. *13*, 442–447.

Santos, M.A., and Tuite, M.F. (1995). The CUG codon is decoded in vivo as serine and not leucine in Candida albicans. Nucleic Acids Res. *23*, 1481–1486.

Schneider, S.U., and de Groot, E.J. (1991). Sequences of two rbcS cDNA clones of Batophora oerstedii: structural and evolutionary considerations. Curr. Genet. *20*, 173–175.

Schneider, S.U., Leible, M.B., and Yang, X.P. (1989). Strong homology between the small subunit of ribulose-1,5-bisphosphate carboxylase/oxygenase of two species of Acetabularia and the occurrence of unusual codon usage. Mol. Gen. Genet. *218*, 445–452.

Schultz, D.W., and Yarus, M. (1994). Transfer RNA mutation and the malleability of the genetic code. J. Mol. Biol. *235*, 1377–1380.

Sengupta, S., and Higgs, P.G. (2015). Pathways of Genetic Code Evolution in Ancient and Modern Organisms. J. Mol. Evol. *80*, 229–243.

Sladic, R.T., Lagnado, C.A., Bagley, C.J., and Goodall, G.J. (2004). Human PABP binds AU-rich RNA via RNA-binding domains 3 and 4. Eur. J. Biochem. *271*, 450–457.

Swart, E.C., Bracht, J.R., Magrini, V., Minx, P., Chen, X., Zhou, Y., Khurana, J.S., Goldman, A.D., Nowacki, M., Schotanus, K., et al. (2013). The Oxytricha trifallax macronuclear genome: a complex eukaryotic genome with 16,000 tiny chromosomes. PLoS Biol. *11*, e1001473.

Tourancheau, A.B., Tsao, N., Klobutcher, L.A., Pearlman, R.E., and Adoutte, A. (1995). Genetic code deviations in the ciliates: evidence for multiple and independent events. EMBO J. *14*, 3262–3267.

Tuite, M.F., and McLaughlin, C.S. (1982). Endogenous read-through of a UGA termination codon in a Saccharomyces cerevisiae cell-free system: evidence for involvement of both a mitochondrial and a nuclear tRNA. Mol. Cell. Biol. *2*, 490–497.

Vallabhaneni, H., Fan-Minogue, H., Bedwell, D.M., and Farabaugh, P.J. (2009). Connection between stop codon reassignment and frequent use of shifty stop frameshifting. RNA *15*, 889–897.

Wang, R., Xiong, J., Wang, W., Miao, W., and Liang, A. (2016). High frequency of +1 programmed ribosomal frameshifting in Euplotes octocarinatus. Sci. Rep. *6*, 21139.

Yang, Z. (2007). PAML 4: phylogenetic analysis by maximum likelihood. Mol. Biol. Evol. *24*, 1586–1591.

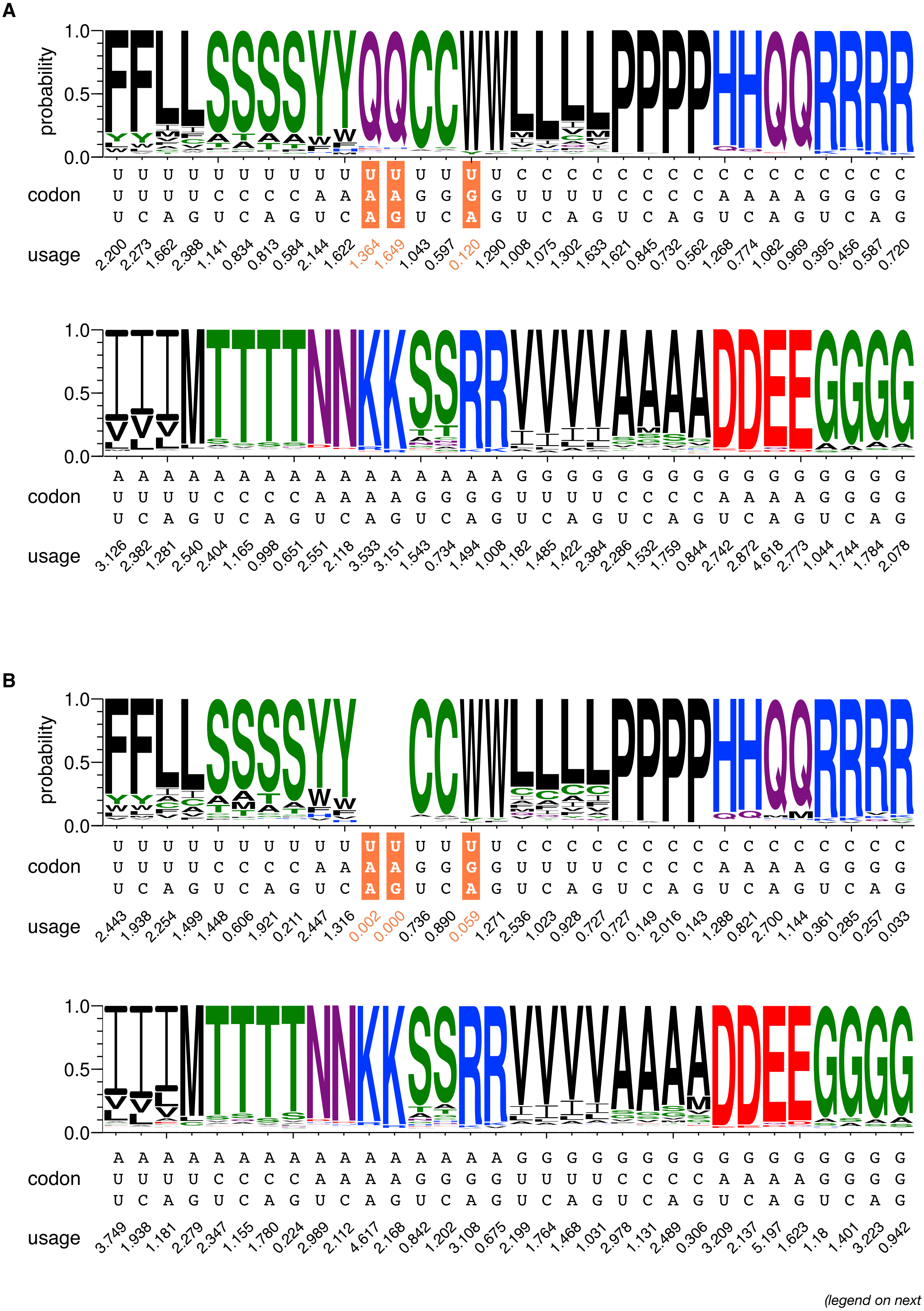

(legend on next page)

Figure S1. Predicted Codon Translations of *Parduczia sp*. and *B. japonicum*, Related to Figure 1

Stop codons in the standard genetic code are highlighted by orange rectangles. Coding sequence codon usage is listed below each codon in percentage.

(A) *Parduczia sp*.

(B) *B. japonicum*. Codon usage for *Parduczia sp*. and heterotrichs is provided in Data S1C.

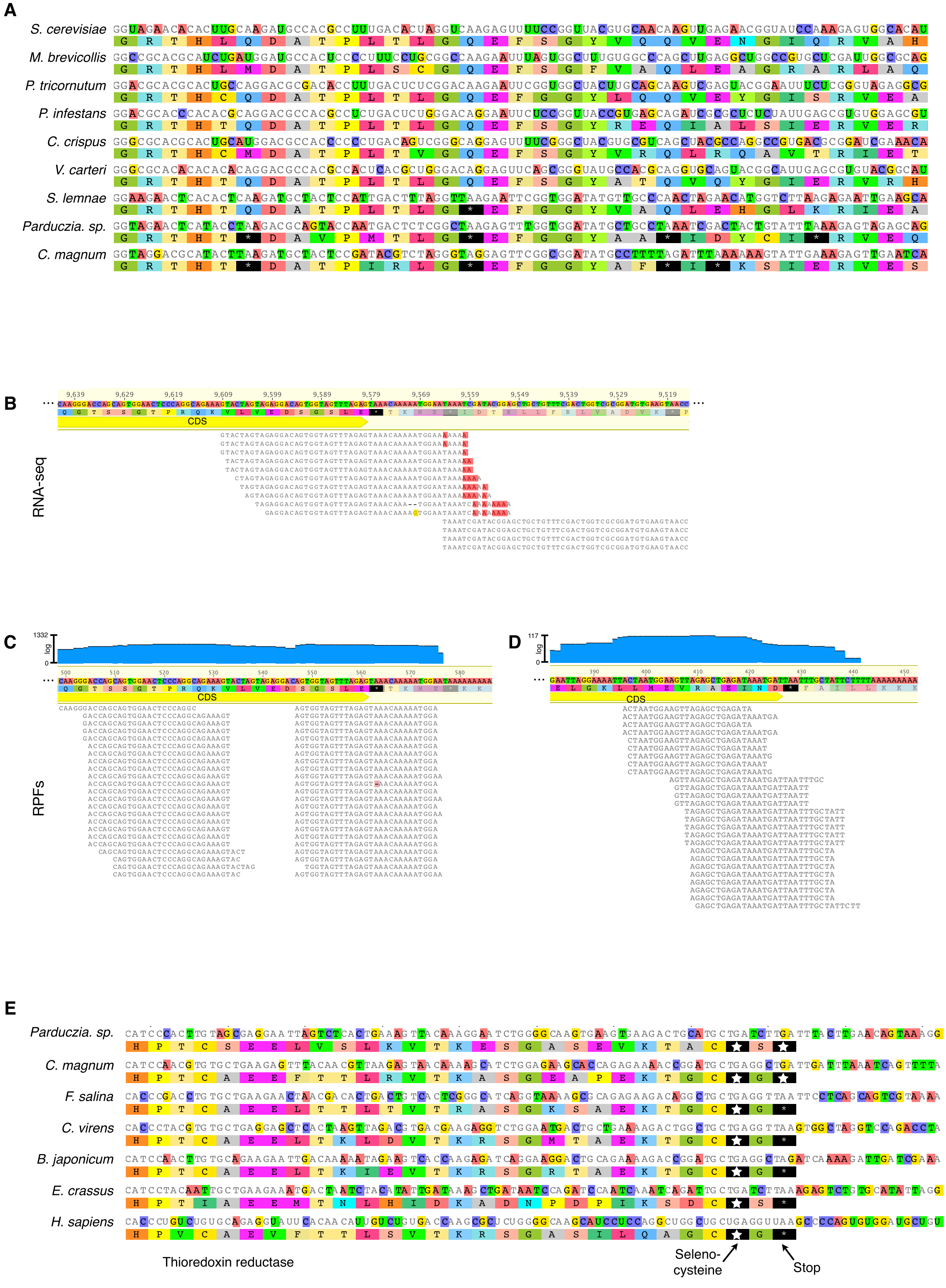

(legend on next page)

Figure S2. Sense and Stop Codons in *C. magnum* and *Parduczia sp.*, Related to Figure 2

(A) Region of a multiple sequence alignment of fumarate hydratase coding sequences highlighting UAA and UAG stop codons. Sequence accessions from GenBank are: NM_001184076 - *S. cerevisiae*; XM_001747580 - *M. brevicolis*; XM_002180443 - *P. tricornutum*; XM_002998645 - *P. infestans*; XM_005717962 - *C. crispus*; XM_002952102 - *V. carteri*; CCKQ01008699 - *S. lemnae*. *Parduczia sp*. and *C. magnum* are transcripts MMETSP1317: CAMNT_0047611615 and MMETSP0210: CAMNT_0008295093, respectively.

(B) A putative UAA terminated gene encoding a cyclophilin protein is shown with mapped poly(A)-tailed reads. Red A's not matching the reference sequence indicate the presence of untemplated poly(A) tails. The yellow arrow indicates a coding sequence (CDS) 3′ end. Note that from multiple sequence alignments alone it is uncertain which of the UAAs after the indicated CDS is a stop. A downstream transcript overlaps with the upstream transcript, but, as indicated by paired-end reads, these transcripts are completely separate (Data S1S and S1T). Left transcript: MMETSP0210: CAMNT_0008294993; contig: 19477__len__16004 is shown; additional UAA ending CDSs are MMETSP0210: CAMNT_0008292199 and MMETSP0210: CAMNT_0008294929 (both CDSs are in the +3 translation frame).

(C) RPFs mapped to the transcript corresponding to a transcript of the gene in (B) showing that termination exclusively occurs at the first of the two UAA codons. This example also shows the characteristic translation terminating RPF 3′ end locations, 11/12 nt downstream of primary UAA stop codon. Light blue graph shows the coverage by RPFs, shown on a log scale. Data S1AM and S1AN.

(D) Ribo-seq read mapping to Trinity transcript c22364_g1_i1. Data S1AO,AP.

(E) Multiple sequence alignment of thioredoxin reductase homologs. MMETSP IDs are MMETSP0210: CAMNT_0008293887 for *C. magnum* and MMETSP1317: CAMNT_0047591293 for *Parduczia sp*.; MMETSP1345: CAMNT_0049039981, MMETSP1397: CAMNT_0052074549, MMETSP1395: CAMNT_0049649177, MMETSP1380: CAMNT_0042421825 for the remaining ciliates; *Homo sapiens* thioredoxin reductase is from GenBank NM_001093771. In mammals and other eukaryotes the penultimate sense codon (1975-1977 in the multiple sequence alignment) encodes a catalytic selenocysteine (Lee et al., 2000). The position of the thioredoxin selenocysteine codon in *C. magnum* and *Parduczia sp*. is shortly before the SECIS element, contrary to a model proposing the necessity of a minimal distance of 51-111 nt between selenocysteine UGA codons and SECIS elements (Martin et al., 1996).

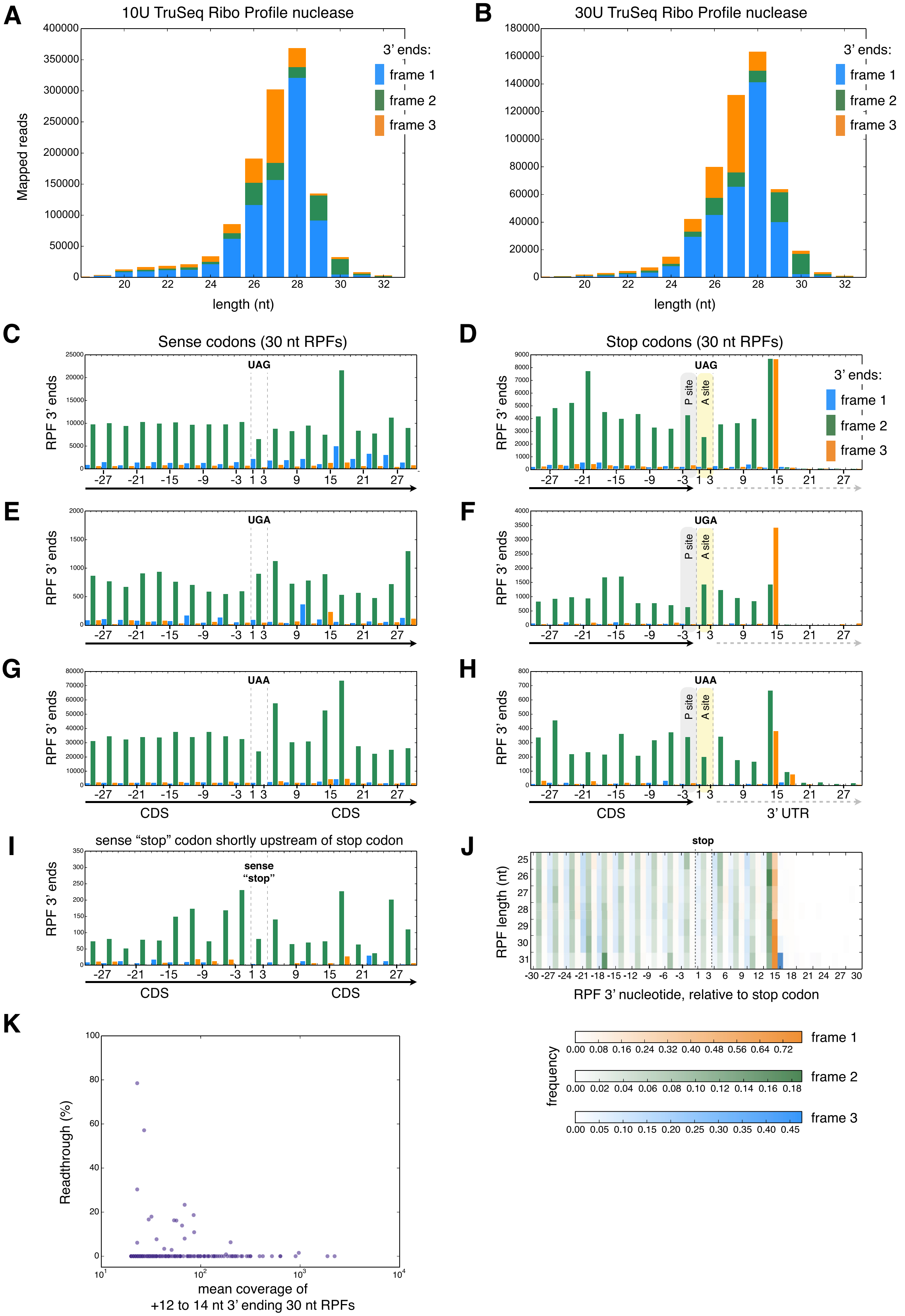

(legend on next page)

Figure S3. Properties of Ribo-seq Data at Sense and Stop Codons, Related to Figure 3

(A and B) Distributions for 10U and 30U of TruSeq Ribo Profile nuclease used to produce RPFs. The peak RPF length is at 28 nt and most RPF 5′ starts and 3′ ends are in frame 1 as for *Saccharomyces cerevisiae* RPFs (Ingolia et al., 2009).

(C–H) Distribution of 30 nt RPFs for individual sense and stop UAG, UGA and UAA codons (positions 1 to 3) in Trinity assembled transcripts.

(I) 30 nt RPF coverage of UAA, UGA and UAA codons located 24-66 nucleotides upstream of their stops.

(J) RPF 3′ end distribution around stop codons for 25-31 nt RPFs; frequencies of RPF ends are calculated for each RPF length.

(K) Stop codon readthrough. See the Supplemental Experimental Procedures for the manner in which readthrough was measured.

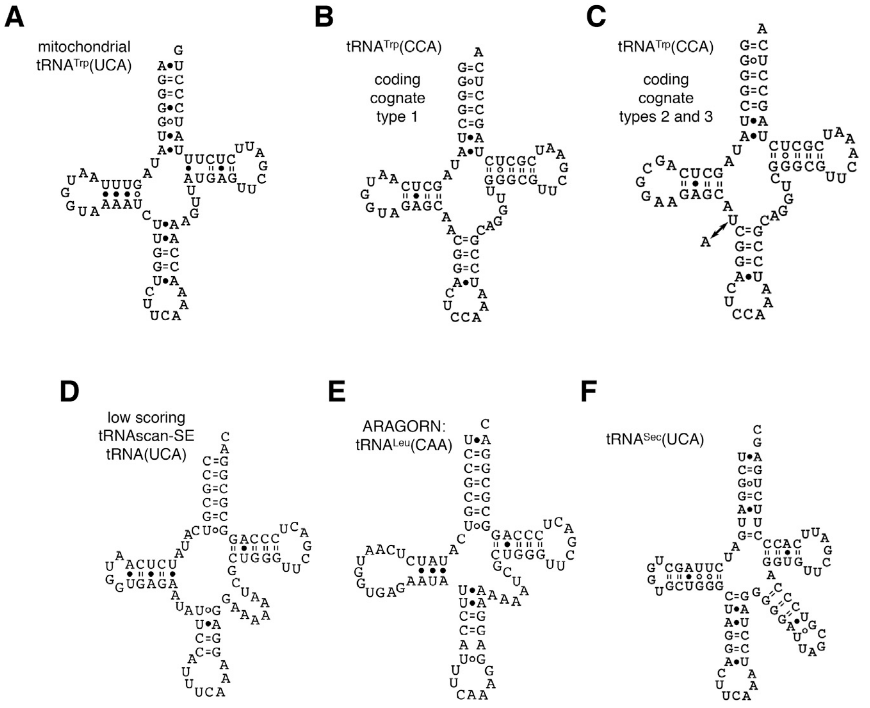

Figure S4. Additional Predicted tRNAs, Related to Figure 4

(A) mitochondrial genome-encoded $tRNA^{Trp}$(UCA) found on Minia assembly mitochondrial contig 3__len__11145 (positions 198-128).

(B) macronuclear genome-encoded tryptophan tRNA found in the Minia assembly

(C) represents two macronuclear genome-encoded tryptophan tRNAs with CCA anticodons with a single base difference between the forms. Judging from our assemblies there may be more than three *C. magnum* $tRNA^{Trp}$(CCA) paralogs.

(D) Predicted tRNA(UCA) with a low tRNAscan-SE score.

(E) Alternative tRNA structure predicted by ARAGORN for the same region as (D). Free energies calculated by RNAeval (default parameters) for the RNAfold centroid structure and the ARAGORN structures for (D) and (E), are -21.3 and -15.6 kcal/mol, respectively.

(F) Selenocysteine tRNA(UCA) found by ARAGORN (Laslett and Canback, 2014). The selenocysteine tRNA is found in the draft *C. magnum* genome assembly contig 24660__len__69094 (positions 7543-7626).

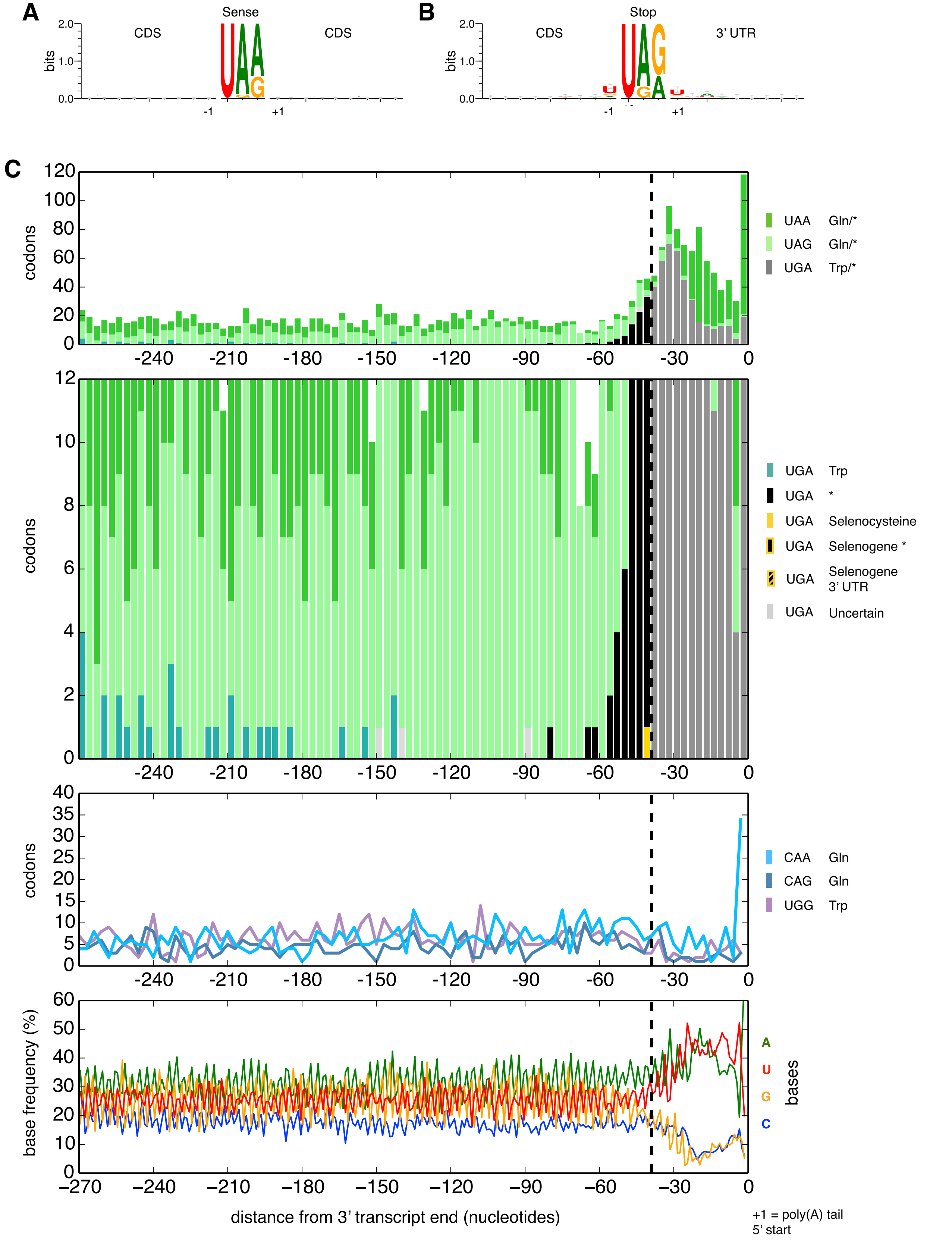

(legend on next page)

Figure S5. Factors Responsible for Discrimination of Stop from Sense, Related to Figure 6

(A) Sequence logos of regions surrounding *C. magnum* UAA, UAG and UGA sense codons. For the central sense codon itself the underlying base frequencies are shown, not bit scores as for the surrounding bases.

(B) Sequence logos of regions surrounding *C. magnum* UAA, UAG and UGA stop codons. For the central stop codon itself the underlying base frequencies are shown.

(C) Graphs like those of Figure 5 for Parduczia sp. Transcript ends begin, and include 0, 1, or 2 nucleotides of the poly(A) tail (position 0) to maintain reading frame. The top two subgraphs showing UAA, UAG and UGA counts are for the same data drawn to different scales.

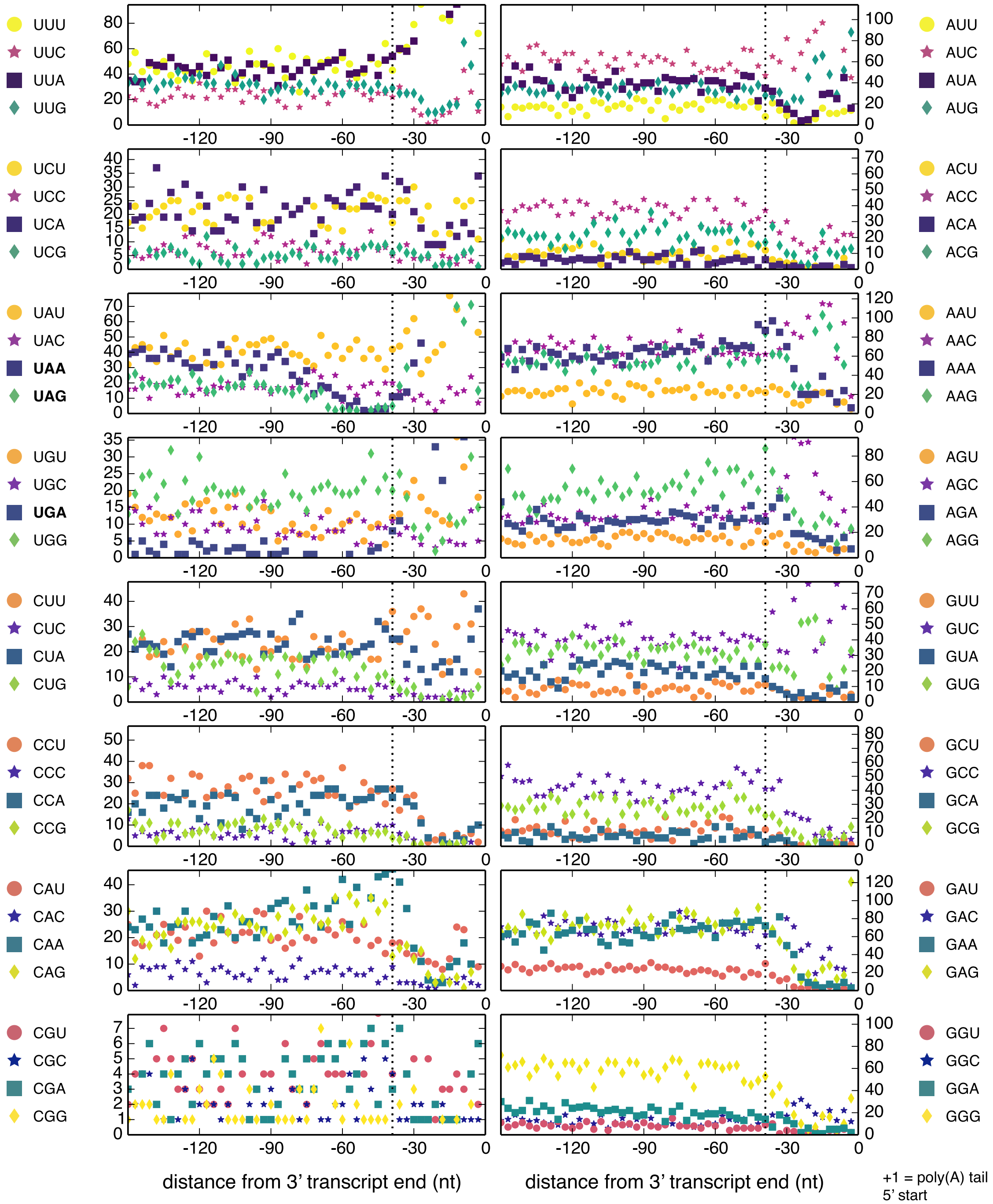

Figure S6. Codon Usage of mRNA 3′ Ends, Related to Figures 5 and 6

Vertical line at 39 nt as in Figure 6 indicates approximately where stop codons begin (hence the frequency of 3′ UTR sequences downstream of this increases up to the poly(A) tail addition site (+1)).

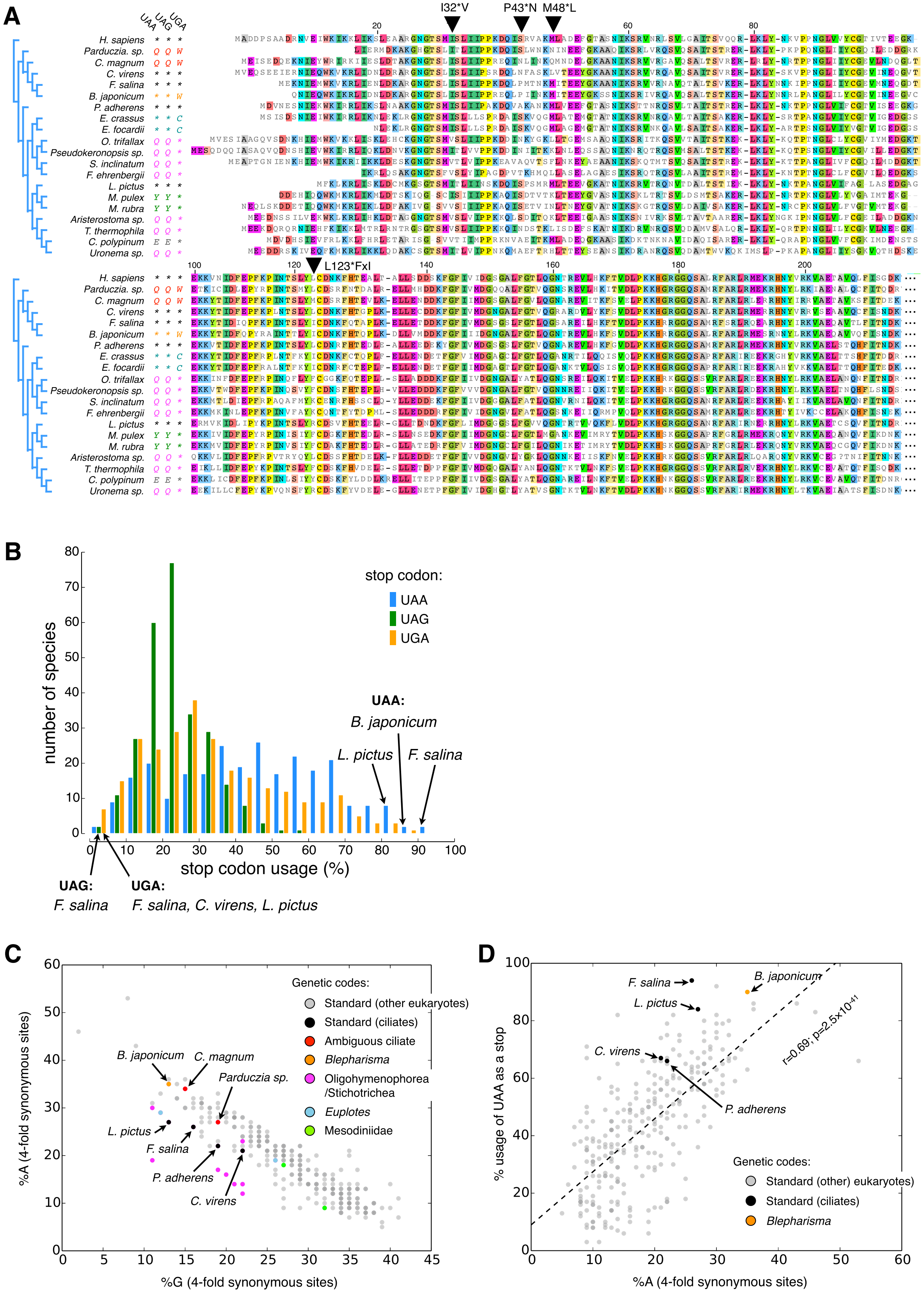

(legend on next page)

Figure S7. Evaluation of Alternative Hypotheses for Stop Codon Reassignments in Ciliates, Related to Figure 1

(A) Multiple sequence alignment underlying the phylogeny in Figure 1A; sequences obtained from UniProt; downloaded Feb 22, 2015. Only the N-terminal half of the alignment is shown. The full alignment can be obtained from Data S1U. Stop codon reassignments are shown to the left of the figure. For clarity ambiguous codons of *C. magnum* and *Parduczia sp*. only have the amino acid reassignment shown. Coordinates are according to those in Lozupone et al., 2001. Sites marked with inverted triangles are those in Lozupone et al., 2001 that were proposed to distinguish the eRF1 of ciliates with UAR or UGA assignments from other eRF1s, and to be sites of convergent evolution between *Stylonychia*/*Oxytricha* and *Tetrahymena* (*) or *Euplotes* and *Blepharisma* (x) (e.g., L123*FxI convergently changed to F in *Stylonychia*/*Oxytricha* and *Tetrahymena* and to I in *Euplotes* and *Blepharisma*). For each of these sites there are exceptions to the hypothesis that convergent amino acid changes in eRF1 have led to the independent evolution of the same genetic codes in different ciliate lineages; for example, L123F substitutions are not found in multiple ciliates with UAR = glutamine reassignments.

(B) Stop codon usage of transcripts ending with poly(A) tails (one transcriptome per species, for species with $\geq$ 50 identified stop codons; see Data S1W for exact values for each species). UGA is rarely a stop in *C. virens* (5%) and *F. salina* (1%), and UAG is rarely a stop in *F. salina* (4%). Ciliates with standard genetic codes from two other classes also have very skewed UAA stop codon usage: 85% in *Litonotus pictus* (class Litostomatea), and 98.5% in *Nyctotheris ovalis* (class Clevelandellida) (Ricard et al., 2008). In *B. japonicum*, which translates UGA as tryptophan (Figure S1B), UAA (91%) is also strongly favored.

(C) Comparison of A and G composition of 4-fold synonymous sites (proxies for neutral site base composition) from ESTScan coding sequence predictions. Each data point represents one MMETSP species. 4-fold synonymous sites for *C. magnum* are 34% A and 15% G; and for *Parduczia sp*., 27% A and 19% G. Note that the transcriptomes underlying the data points are a combination of ciliate transcripts and transcripts from other sources (e.g., ciliate food); the latter transcripts typically originate from more GC rich genomes and so deflate the %A and inflate the %G of the ciliate 4-fold sites. In certain transcriptomes, e.g., those belonging to the genus *Mesodinium*, a large proportion of the transcripts are of non-ciliate origin (indicated in Table S1).

(D) Relationship of UAA usage to 4-fold synonymous site A usage. A linear regression is indicated with a dashed line, with its correlation coefficient and the two-tailed p value for testing with the null hypothesis of a regression slope of zero below the line. Ciliates with variant codes, other than *Blepharisma japonicum*, are not indicated because their codes lead to widespread mispredictions of stop codons by ESTScan (in the case of *B. japonicum*, we removed all the predictions with UGA stops as none of the stops appeared to be genuine).

Cell, Volume *166*

Supplemental Information

Genetic Codes with No Dedicated Stop Codon:

Context-Dependent Translation Termination

Estienne Carl Swart, Valentina Serra, Giulio Petroni, and Mariusz Nowacki

Supplemental Experimental Procedures

Condylostoma magnum culturing

Condylostoma magnum strain COL2 (500-550 µm longest dimension) was isolated in 2007 as a single cell from rocky seaside pools near the Accademia Navale in Livorno, Italy. Cells were kept at room temperature and fed regularly with *Dunaliella tertiolecta* and occasionally with *Phaeodactylum tricornutum.* To grow *D. tertiolecta* and *P. tricornutum*, a saline solution was made with the following: 37 g of Red Sea Salt (company: Red Sea), 1 ml of Walne's solution (Walne, 1970) and 1 drop of multivitamin complex B (B1, B6, and B12; Bayer Benexol B12) made up to 1L with distilled water. Walne's solution was prepared as: 100 g $NaNO_3$, 45 g EDTA (disodium salt), 33.6 g H_3BO_3, 20 g $NaH_2PO_4.H_20$, 0.36 g $MnCl_2$, 1.8 g EDTA (disodium salt), 1 ml of TMS (Trace Metal Solution: 2.1 g $ZnCl_2$, 2.0 g $CoCl_2.6H_2O$, 0.9 g $(NH_4)_6Mo_7O_{24}.4H_2O$, 2.0 g $CuSO_4.5H_2O$, H_2O to 100 ml; acidified with a few drops of concentrated HCl to clarity), made up to 1 L in distilled water. The microalgal culture was incubated at 19°C with a 12 h light/dark cycle (Osram Daylight lamp, 36W/10 and Osram Fluora lamp, 40W/77).

Checks for potential contaminating transcripts

To check for possible contaminants (e.g. from the algal food source, *Pheodactylum tricornutum*) among the assembled *C. magnum* transcripts we examined sequence base composition and assembled rRNAs (MMETSP: CAMNT_0008266115, CAMNT_0008312561). Base composition is unimodal (mode 33% GC) and no rRNAs other than from *C. magnum* were found, suggesting that this data is predominantly comprised of *C. magnum* transcripts. *C. magnum*'s food source, the diatom *P. tricornutum* (European Nucleotide Archive (ENA): GCA_000150955.2), has transcripts that are more GC rich (mode 50% GC) than those of *C. magnum*, and has the standard genetic code (Data S1F). As evidenced by the absence of Pfam domains (Finn et al., 2014) matching typical mitochondrially-encoded ciliate proteins (e.g. COX1, COX2, COB, NAD5) during HMMER3 (Eddy, 2014) searches (default parameters, independent e-value < 1e-3) of the assembled *C. magnum* RNA-seq data, mitochondrial transcript levels are negligible.

Shotgun proteomics and verification of translation of UGA as tryptophan

200 µl of *C. magnum* cells (> 30,000 cells) were lysed in 1 ml of protein loading buffer (from a 10 ml stock of 2 ml 10% SDS; 1.2 ml 0.5 M Tris-Cl pH 6.8; 4.8 ml 50% glycerol; 1.2 mg Bromophenol blue; 500 µl β-Mercaptoethanol; 1.5 ml ddH_20) and stored at -20˚C until further processing. 5 µl of this sample was incubated at 95˚C for 5 minutes. SDS-PAGE was used to separate the proteins with a 5% stacking gel (2.2 ml ddH_20; 0.67 ml 30% acrylamide/Bis solution (Bio-Rad); 1 ml Tris-Cl (0.5 M, pH 6.8); 0.04 ml 10% SDS; 0.04 ammonium persulfate; 4 µl TEMED) on top of a 10% resolving gel (5.9 ml ddH_20; 5.0 ml 30% acrylamide/Bis solution (Bio-Rad); 3.8 ml Tris-Cl (0.5 M, pH 6.8); 0.15 ml 10% SDS; 0.15 ammonium persulfate; 6 µl TEMED) an electrophoresis buffer (3.2 g Tris; 14.4 g glycine; 1 g SDS made up to 1 L with ddH_20). After staining the gel with InstantBlue (Expedeon) and destaining in a 10% acetic acid, 25% methanol solution, 10 slices of ~1 mm width were cut from the resolving gel, and diced into six ~1 mm^3 cubes which were stored in 20% ethanol at -20˚C before further processing.

Gel cubes were washed with 50 mM Tris/HCl pH 8 (Tris buffer) and Tris buffer/acetonitrile (LC-MS grade, Fluka, Buchs, Switzerland) 50/50 before protein reduction with 50 mM DTT (Fluka, Buchs, Switzerland) in Tris buffer for 30 min at 37°C, and alkylation with 50 mM iodoacetamide (Fluka, Buchs, Switzerland) in Tris buffer for 30 min at 37°C in the dark. The gel cubes were then soaked with trypsin solution (10 ng/ml trypsin (Promega) in 20 mM Tris/HCl pH 8, 0.01% ProteaseMax (Promega)) for 30 min on ice, then covered by 5–10 ml 20 mM Tris/HCl before digestion for 60 min at 50°C. A 5 µl injection of the protein digest was then analyzed by liquid chromatography-tandem mass spectrometry (LC)-MS/MS (DIONEX Ultimate coupled to a QExactive mass spectrometer, ThermoFisher Scientific). Peptides were trapped on an Acclaim PepMap100 C18 pre-column (3 µm, 100 Å, 75 µm×2 cm, ThermoFisher Scientific, Reinach, Switzerland) and separated by backflush on a C18 column (5 µm, 100 Å, 75 µm×15 cm, Magic C18) by applying a 60 minute gradient of 5% to 40% acetonitrile in water and 0.1% formic acid, at a flow rate of 400 nl/min. The full-scan method was set with resolution at 70,000 with an automatic gain control (AGC) target of 1e06 and maximum ion injection time of 50 ms. The data-dependent method for precursor ion fragmentation was applied with the following settings: resolution 17,500, AGC of 1e05, maximum ion time of 110 milliseconds, mass window 2 *m*/*z*, collision energy 27, underfill ratio 1%, charge exclusion of unassigned and 1+ ions, and peptide match preferred, respectively. A database of six-frame translations of the *C. magnum* transcriptome assembly, translated with UAR=glutamine, UGA=tryptophan, was used as input for *in silico* peptide fragmentation and peptide identification by EasyProt (Gluck et al., 2013) (default parameters: 1% false discovery rate and two peptides for acceptance of a protein identification).

To verify tryptophan translation at UGA codons, we examined each of the peptides identified by mass spectrometry containing a tryptophan translated from a UGA codon (25 total; Data S1D). 22 of these peptides were in the same reading frame as the best BLASTX match of their transcript to *O. trifallax* predicted proteins (using the BLAST server at oxy.ciliate.org (Stover et al., 2012; Swart et al., 2013); e-value < 1e-6), and each of the remaining three peptides had no BLASTX matches to the transcript from which it was derived.

Codon usage prediction

To determine codon usage (Figure 1B and Figure S1) we extracted best BLASTX matching regions (-query_gencode 6; e-value < 1e-20) of coding sequences from poly(A) tailed MMETSP transcripts used as queries to an *O. trifallax* predicted protein database (Swart et al., 2013). For ciliates with the standard genetic code (e.g. the heterotrichs *Climacostomum virens* and *Fabrea salina*), our codon usage estimates for UAA, UAG and UGA codons are 0-0.008%, and appear to represent algorithmic errors (e.g. "stops" located close to the ends of BLAST matches, which may occur when the local alignment extends beyond true protein ends), and selenocysteine codons. Codon usage of the ciliates examined in this manuscript is provided as Data S1C.

Stop codon and 3' UTR identification

To predict the stops of *C. magnum* coding sequences in poly(A)-terminated transcripts (possessing a terminal poly(A) ≥ 7 nt), we visually inspected BLASTX (Camacho et al., 2009) results of *C. magnum* transcripts vs. proteins from *O. trifallax* (Swart et al., 2013) (local BLASTX; best BLASTX match; e-value < 1e-6; query genetic code 6); *T. thermophila* (Stover et al., 2012) and GenBank's nr (nonredundant) database were also used when there was uncertainty about the match ends from the *O. trifallax* BLASTX matches. The stop codon was chosen as the first UAA, UAG, or UGA codon downstream of the BLASTX match closest to the C-terminal end of the *O. trifallax* or *T. thermophila* proteins. Sequences where the top matches terminated close (~6 amino acids) to the predicted query stop codons were then selected. This procedure annotated 150 putative *C. magnum* 3' UTRs, and the coding sequence (CDS) regions upstream to the beginning of the best BLASTX matches (Data S1R). With the appropriate query genetic code and stop codons, this procedure was also used to annotate 50 3' UTRs from *B. japonicum*, *Climacostomum virens*, *Euplotes crassus* and *Pseudokeronopsis sp.,* and 70 3' UTRs from *Parduczia. sp.* (Data S1R). Note that UAA terminated coding sequences are underrepresented in the resultant *C. magnum* data set (0% of transcripts) relative to those automatically inferred from the Trinity transcriptome (11%) described in the next paragraph. In our inspection of *Parduczia sp.* transcripts (by means of BLASTX matches to ciliates and other eukaryotes) we found no examples of coding sequences terminated by either UAG or UAA as stops.

To generate larger data sets to analyze stop codons, Trinity (default parameters) was used to asssemble new transcriptomes from both the *C. magnum* and *Parduczia sp*. MMETSP RNA-seq data (Data S1X and Data S1Y, respectively). BLASTX searches (best matches; e-value < 1e-20; query genetic code 6) of poly(A)-tailed transcripts from *C. magnum* and *Parduczia sp.* vs. *O. trifallax* proteins were used to infer reading frame, excluding cases where the BLAST match was to the reverse complement of the strand possessing the poly(A). Poly(A) tails were trimmed down to 0, 1 or 2 nucleotides to maintain the reading frame when counting codons back from the transcript ends (position 0 in Figure 6; Figure S5 and S6). From these transcripts, single gene (Trinity classification), single isoform transcripts were selected for "stop" codon and ribo-seq analysis (yielding 1672 transcripts for *C. magnum* and 455 for *Parduczia sp.*; Data S1Z and S1AA).

Based on our analysis of the 3' UTR length distributions of the curated MMETSP transcripts, we generated a data set of transcripts with only a single possible stop in the region 60 nt upstream of the poly(A)-tailed Trinity assembled transcripts (excluding the poly(A) tail length), yielding 294 transcripts (Data S1AB). For *C. magnum* transcripts with only a single possible stop, the frequencies of UAG, UGA and UAA stops are 62%, 25% and 13%, respectively. Scanning downstream from 60 nt upstream of the poly(A) tail to identify the first downstream "stop" codon, i.e. the putative primary stop, 1378 (82%) transcripts have additional possible stop codons downstream of the putative primary stop. As judged from ribosome profiling data, this procedure correctly classifies the bulk of primary stop codons (with little readthrough; Figure 3D and Figure S3D-H). The overall length distribution of the 3' UTRs downstream of the Trinity transcript putative primary stops is similar to that of the manually curated 3' UTRs (peaking around 18-21 nt). 12 of 39 (31%) 3' UTRs with UAA as the primary stop are of length 0. For zero nt 3' UTRs, 8 of 12 are consistent with the positions of stops in other organisms, as judged by BLASTX searches, and/or by RPFs ending 11/12 nt downstream of the UAA (compared to 17 of the 27 3' UTRs > 0 nt long assessed by the same criteria); no evidence suggests that the remaining four zero nt 3' UTRs are incorrect predictions.

Stop codon readthrough detection and estimation

The fraction of readthrough, $r = cov_{stop} \div (cov_{stop} + cov_{downstream})$, is measured relative to the stop (positions +1 to +3) for transcripts with at least twenty 30 nt RPF 3' ends at positions +12 to +14 (the positions corresponding to the characteristic termination signal, as in Figure 3D). cov_{stop} = (30 nt RPF 3' end counts at positions +12 to +14) ÷ 3; $cov_{downstream}$ = (30 nt RPF 3' end counts from positions +17 to the 3' UTR end, excluding the poly(A) tail) ÷ (number of positions in 3' UTR at which 30 nt 3' RPF ends were counted). Note that due to the counting of only covered positions in the denominator of $cov_{downstream}$, readthrough will be overestimated (if all the positions in the 3' UTR were used instead, readthrough would be underestimated). Transcripts are considered to be read through if $r > 0$.

Stop codon usage estimation

For the 670 MMETSP transcriptomes we downloaded, stop codon usage was estimated for poly(A)-ending (≥ 7 nt) contigs from the MMETSP ESTScan (Iseli et al., 1999) coding sequence predictions (Keeling et al., 2014) ending on TAA, TAG, or TGA. Transcriptomes with ≥ 50 putative stop codons were used for stop codon usage estimation, excluding ciliates with non-standard

genetic codes except *B. japonicum*. Note that UAA stop codon usage is underestimated for transcriptomes with a significant proportion of non-ciliate transcripts (often originating from the sources indicated in Table S1). A table of stop codon counts for these transcriptomes is provided in Data S1W.

Sequence logos of regions flanking *C. magnum* sense and stop codons

Sequence logos were created with WebLogo 3.3 (Crooks et al., 2004). For 3' UTRs, sequence logos use a compositional adjustment of 3' UTR base frequencies, excluding the stop codon and poly(A) tail (e.g. for *C. magnum*: A=39%, C=5%, G=13%, U=43%). For coding sequence (CDS) positions immediately upstream of the stop, a compositional adjustment is done frame-wise for each of the three reading frames, based on manually curated coding sequence base frequencies (frame 1: A=32%, C=14%, G=33%, U=21%; frame 2: A=35%, C=19%, G=18%, U=28%; frame 3: A=31%, C=16%, G=19%, U=33%).

Multiple sequence alignments

MAFFT 7.017 (Katoh and Standley, 2013) was used for all multiple sequence alignments. The default parameters in Geneious 7.1.4 (Kearse et al., 2012) were used for both alignment methods.

d_N/d_S estimation

d_N/d_S values were estimated using codeml version 4.7b (Yang, 2007) for a pairwise alignment of *C. magnum* tryptophan-tRNA ligase (CAMNT_0008287141) and *Oxytricha* tryptophan-tRNA ligase (GenBank: EJY83191.1).

Genome sequencing, assembly and read mapping

A NucleoSpin Plant II kit (MACHEREY-NAGEL) was used to isolate total DNA from a 0.1 ml of *C. magnum* cells pelleted from a 2 L culture. We assembled a draft *C. magnum* macronuclear genome using the Minia genome assembler (Chikhi and Rizk, 2013) with default parameters and a k-mer size of 85. An additional assembly produced by the IDBA_UD assembler (Peng et al., 2012) was also examined in some cases (e.g. tRNA searches). Over the larger *C. magnum* contigs we examined, we observe reasonably even sequence coverage (mean ~140×). As is typical of ciliates, *C. magnum* has a micronuclear genome in addition to its macronuclear genome. In this assembly, micronuclear sequences are likely to be minimal since macronuclear DNA is typically highly amplified in large ciliates (> 1000×) (Prescott, 1994). We were also able to assemble a large portion (29.9 kb) of the *C. magnum* mitochondrial genome (contigs 3__len__11145, 0__len__6198, 1__len__11219 and, 2__len__1536; identified by BLAST searches vs. other ciliate mitochondrial genomes). Due to *C. magnum*'s unusual genetic code, an accurate automated gene prediction method still needs to be developed, and so we provide just the raw macronuclear genome assembly at present (European Nucleotide Archive accession: ERS696421). Paired-end reads were mapped to the draft *C. magnum* assembly using BWA (Li and Durbin, 2009) (default parameters).

Computational tRNA identification

tRNAscan-SE (Lowe and Eddy, 1997) with default parameters was initially used to predict tRNAs in the *C. magnum* Minia genome assembly. In our draft *C. magnum* genome assembly tRNAscan-SE did not detect a selenocysteine tRNA, but ARAGORN (default parameters) did (Figure S4F).

BLASTN searches (word size of 4) of the draft *C. magnum* genome detected no additional paralogs of $tRNA^{Trp}$(CCA) beyond those identified by tRNAscan-SE. No reads among those mapped to *C. magnum* $tRNA^{Trp}$(CCA) genes with STAR ((Dobin et al., 2013); default parameters) suggested the presence of unassembled sequences from undetected close tryptophan tRNA paralogs. ARAGORN (Laslett and Canback, 2004) searches (default parameters) found no *C. magnum* $tRNA^{Trp}$(UCA)'s at the read level, other than the mitochondrial and selenocysteine tRNAs (Figure S4A and S4F, respectively; Data S1H).

Reducing tRNAscan-SE's Cove cutoff score to 10 allowed the discovery of a single putative tRNA(UCA) with a Cove score of 11.51 (Figure S4D; Data S1G; on contig 14671__len__38937). This tRNA has an unusual eight base anticodon with a potential UCA anticodon complementary to the UGA codon and falls in a region with no mapped MMETSP RNA-seq reads (using STAR; Data S1I,J). For the same sequence plus one base, a leucine tRNA with a CAA anticodon is predicted by ARAGORN (Laslett and Canback, 2004) (Figure S4E; default parameters), making the anticodon recognized by the lower scoring tRNAscan-SE prediction doubtful. Only one other tRNA (Cove score 12.54) was found below the default scoring threshold of 20, and had no possible UCA anticodon. This tRNA also falls in a region with no mapped MMETSP RNA-seq reads (Data S1M,N). Expression of these candidate tRNAs is supported by tRNA-derived sRNA-seq reads, including reads with CCA tails characteristic of mature tRNAs (see next section; Data S1K,N).

sRNA-seq for tRNA identification and searches for $tRNA^{Trp}$(CCA) anticodon editing

Total RNA was isolated from > 1000 cells using an miRNAeasy Mini kit (Qiagen). 60-100 nt RNAs were size-selected on an electrophoretic gel and paired-end RNA-seq libraries (125 bp reads, only one direction was provided) were prepared using standard Illumina protocols by Fasteris (Geneva, Switzerland). After quality control and adaptor trimming by Fasteris there were 1.9 million 10-99 bp reads. Raw reads were deposited in the European Nucleotide Archive (accession numbers: ERS744875 and

ERS744876). 30-89 bp reads were mapped to the entire tRNA-encoding contigs using BWA (Li and Durbin, 2009) with default parameters.

To facilitate ease of viewing we extracted all the reads mapping to $tRNA^{Trp}$(CCA) genes in our assembly and mapped them back to a representative tRNA on contig 27450__len__809 with the Geneious read mapper and default maximum sensitivity parameters (Data S1P). None of the 164 reads mapping through or up to the first codon of the $tRNA^{Trp}$(CCA) anticodons had evidence of 1st position anticodon C→U editing (Data S1P,Q).

Searches for $tRNA^{Trp}$(CCA) anticodon editing by RT-PCR and Sanger sequencing
To search for CCA→UCA anticodon editing we performed either single cell RT-PCR (Trp1 and Trp2 primer combinations described later in this paragraph) or RT-PCR on RNA isolated from 15 *C. magnum* cells with the miRNAeasy Mini kit (Qiagen) in 30 ul of nuclease free water (Trp2_f and Trp2_rM primer). Single cells were isolated with a Gilson pipette and washed several times in saline water (33 g/L NaCl) followed by a single wash in distilled water. Individual cells in 2 ul of water, or 2 ul of purified RNA, were combined with 1 ul of reverse transcription primers, 1 ul dNTPs and 6 ul of nuclease free water. Cells were lysed at 65°C for 5 min. Reverse transcription was performed using Superscript III reverse transcriptase (Life Technologies). The following primers were used (primer names with "_r" suffixes were used for cDNA synthesis): Trp1_f: GGGGCTATAGCTCAGCGGAAG, Trp1_r: GTGAGGCTAGAGCGATTTGAACG, Trp2_f: GGGGCTATAGCTCAATGGTAGAG, Trp2_r: GTGAGGCTAGAGCGATTCGAAC, Trp2_rM: TGGTGAGGCTAGAGCGATTC. PCR products purified with the Wizard SV Gel and PCR Cleanup Kit (Promega) were cloned into pGEM-T easy vectors (Promega). Plasmids containing the RT-PCR products were isolated with the Wizard Plus SV Miniprep DNA Purification kit (Promega) before being Sanger sequenced by Microsynth (Switzerland).

In total 77 $tRNA^{Trp}$ sequences were obtained (20 - "Trp1" and 37 - "Trp2" and 20 – “Trp2M”; Data S1O). Just one of 77 sequenced clones had a C→T substitution at the 1st anticodon position (Data S1O,Q); however, since none of the 164 reads obtained from Illumina sRNA-seq had this substitution it seems more likely that it is an RTase or PCR error rather than a genuine editing event.

Detection of selenocysteine UGA codons
In Figure 6 and Figure S5 counts of a few UGA codons in selenogenes can be seen. The *C. magnum* and *Parduczia sp.* transcriptomes both encode selenogenes with the necessary SECIS (selenocysteine insertions sequence) elements for selenocysteine translation. As an example of these selenogenes, both transcriptomes encode a thioredoxin gene whose single catalytic selenocysteine is encoded by a UGA codon, just one codon upstream of its stop (also UGA; Figure S2E).

eRF1 phylogeny
To create an eRF1 phylogeny 16 representitive, manually annotated ciliate eRF1 protein sequences from MMETSP transcripts were chosen. Two additional predicted protein sequences for *Oxytricha trifallax* and *Tetrahymena thermophila*, were obtained from www.ciliate.org, and a human eRF1 protein sequence was obtained from UniProt (accession:P62495). *Carchesium polypinum* eRF1 was obtained from a manually annotated transcript from its Trinity transcriptome assembly. All the eRF1 sequences were then aligned using MAFFT (default parameters in Geneious 7.1.4). This alignment can be seen in Figure S7. To produce the phylogeny, a conserved block of 429 amino acids was manually selected as the input alignment for PhyML (Guindon et al., 2010) (substitution model LG, 100 bootstrap replicates, default parameters in Geneious 7.1.4).

Supplemental Tables

Table S1. Ciliate genetic codes. Related to Figure 1.

ID	"Stop" assignments			Class	Family	Bionomial name	Strain	Food/host	Contami -nation
	UAA	UAG	UGA						
MMETSP0127	*	*	*	Colopdea	Platyophryidae	Platyophrya macrostoma	WH	Bodo caudatus, Enterobacter aerogenes	yes
MMETSP1317	Q	Q	W/*	Karyorelictea	Geleiidae	Parduczia sp.	NA	?	no
MMETSP1395	*	*	W	Heterotrichea	Blepharismidae	Blepharisma japonicum	Stock R1072	bacteria	low
MMETSP1345	*	*	*	Heterotrichea	Climacostomidae	Fabrea salina	Unknown	bacteria	low
MMETSP1397	*	*	*	Heterotrichea	Climacostomidae	Climacostomum virens	Stock W-24	bacteria	no
MMETSP0210	Q/*	Q/*	W/*	Heterotrichea	Condylostomatidae	Condylostoma magnum	COL2	Phaeodactylum tricornutum	no
MMETSP0209	*	*	*	Litostomatea	Litonotidae	Litonotus pictus	P1	Euplotes crassus	no
MMETSP0467	Y	Y	*	Litostomatea	Mesodiniidae	Mesodinium pulex	SPMC105	Heterocapsa rotundata MMETSP0503	yes
MMETSP0798	Y	Y	*	Litostomatea	Mesodiniidae	Mesodinium rubra	CCMP2563	Geminigera cryophila	yes
MMETSP1018	Q	Q	*	Oligohymenophorea	Orchitophryidae	Anophryoides haemophila	AH6	lobster	no
MMETSP1019	Q	Q	*	Oligohymenophorea	Orchitophryidae	Anophryoides haemophila	AH6	lobster	low
MMETSP0125	Q	Q	*	Oligohymenophorea	Unknown	Aristerostoma sp.	ATCC 50986	Klebsiella, other bacteria	no
MMETSP0018	Q	Q	*	Oligohymenophorea	Uronematidae	Uronema sp.	Bbcil	?	no
MMETSP0472	Q	Q	*	Prostomatea	Colepidae	Tiarina fusus	LIS	Rhodomonas lens	yes
MMETSP0205	*	*	C	Spirotrichea	Euplotidae	Euplotes focardii	TN1	Dunaliella tertiolecta	low
MMETSP0206	*	*	C	Spirotrichea	Euplotidae	Euplotes focardii	TN1	Dunaliella tertiolecta	yes
MMETSP0213	*	*	C	Spirotrichea	Euplotidae	Euplotes harpa	FSP1.4	Dunaliella salina and Dunaliella tertiolecta	no
MMETSP1380	*	*	C	Spirotrichea	Euplotidae	Euplotes crassus	CT5	Dunaliella tertiolecta	low
MMETSP0216	*	*	*	Spirotrichea	Protocruziidae	Protocruzia adherens	Boccale	Dunaliella tertiolecta	no
MMETSP0211	Q	Q	*	Spirotrichea	Pseudokeronopsidae	Pseudokeronopsis sp.	OXSARD2	Phaeodactylum tricornutum	?
MMETSP1396	Q	Q	*	Spirotrichea	Pseudokeronopsidae	Pseudokeronopsis sp.	Brazil	bacteria, Phaeodactylum tricornutum	no
MMETSP0123	Q	Q	*	Spirotrichea	Ptychocylididae	Favella ehrenbergii	Fehren 1	Heterocapsa triquetra, Mantoniella squamata, Isochrysis galbana	no
MMETSP0434	Q	Q	*	Spirotrichea	Ptychocylididae	Favella taraikaensis	Fe Narragansett Bay	Heterosigma akashiwo CCMP3107	no
MMETSP0436	Q	Q	*	Spirotrichea	Ptychocylididae	Favella taraikaensis	Fe Narragansett Bay	Heterocapsa triquetra, CCMP 448	no
MMETSP0208	Q	Q	*	Spirotrichea	Strombidiidae	Strombidium inclinatum	S3	Dunaliella tertiolecta	no
MMETSP0449	Q	Q	*	Spirotrichea	Strombidiidae	Strombidium rassoulzadegani	ras09	Tetraselmis chui PLY429	no
MMETSP0126	Q	Q	*	Spirotrichea	Strombidinopsidae	Strombidinopsis acuminatum	SPMC142	Heterocapsa triquetra, Rhodomonas sp. (CCMP 755), Mantoniella squamata, Isochrysis galbana	yes
MMETSP0463	Q	Q	*	Spirotrichea	Strombidinopsidae	Strombidinopsis sp.	SopsisLIS2011	Rhodomonas lens	low

Supplemental References

Camacho, C., Coulouris, G., Avagyan, V., Ma, N., Papadopoulos, J., Bealer, K., and Madden, T.L. (2009). BLAST+: architecture and applications. BMC bioinformatics *10*, 421.

Chikhi, R., and Rizk, G. (2013). Space-efficient and exact de Bruijn graph representation based on a Bloom filter. Algorithms for molecular biology : AMB *8*, 22.

Crooks, G.E., Hon, G., Chandonia, J.M., and Brenner, S.E. (2004). WebLogo: a sequence logo generator. Genome research *14*, 1188-1190.

Dobin, A., Davis, C.A., Schlesinger, F., Drenkow, J., Zaleski, C., Jha, S., Batut, P., Chaisson, M., and Gingeras, T.R. (2013). STAR: ultrafast universal RNA-seq aligner. Bioinformatics *29*, 15-21.

Finn, R.D., Bateman, A., Clements, J., Coggill, P., Eberhardt, R.Y., Eddy, S.R., Heger, A., Hetherington, K., Holm, L., Mistry, J., *et al.* (2014). Pfam: the protein families database. Nucleic acids research *42*, D222-230.

Gluck, F., Hoogland, C., Antinori, P., Robin, X., Nikitin, F., Zufferey, A., Pasquarello, C., Fetaud, V., Dayon, L., Muller, M., *et al.* (2013). EasyProt--an easy-to-use graphical platform for proteomics data analysis. Journal of proteomics *79*, 146-160.

Guindon, S., Dufayard, J.F., Lefort, V., Anisimova, M., Hordijk, W., and Gascuel, O. (2010). New algorithms and methods to estimate maximum-likelihood phylogenies: assessing the performance of PhyML 3.0. Systematic biology *59*, 307-321.

Iseli, C., Jongeneel, C.V., and Bucher, P. (1999). ESTScan: a program for detecting, evaluating, and reconstructing potential coding regions in EST sequences. Proceedings / International Conference on Intelligent Systems for Molecular Biology ; ISMB International Conference on Intelligent Systems for Molecular Biology, 138-148.

Katoh, K., and Standley, D.M. (2013). MAFFT multiple sequence alignment software version 7: improvements in performance and usability. Molecular biology and evolution *30*, 772-780.

Kearse, M., Moir, R., Wilson, A., Stones-Havas, S., Cheung, M., Sturrock, S., Buxton, S., Cooper, A., Markowitz, S., Duran, C., *et al.* (2012). Geneious Basic: an integrated and extendable desktop software platform for the organization and analysis of sequence data. Bioinformatics *28*, 1647-1649.

Laslett, D., and Canback, B. (2004). ARAGORN, a program to detect tRNA genes and tmRNA genes in nucleotide sequences. Nucleic acids research *32*, 11-16.

Li, H., and Durbin, R. (2009). Fast and accurate short read alignment with Burrows-Wheeler transform. Bioinformatics *25*, 1754-1760.

Lowe, T.M., and Eddy, S.R. (1997). tRNAscan-SE: a program for improved detection of transfer RNA genes in genomic sequence. Nucleic acids research *25*, 955-964.

Peng, Y., Leung, H.C., Yiu, S.M., and Chin, F.Y. (2012). IDBA-UD: a de novo assembler for single-cell and metagenomic sequencing data with highly uneven depth. Bioinformatics *28*, 1420-1428.

Prescott, D.M. (1994). The DNA of ciliated protozoa. Microbiological reviews *58*, 233-267.

Stover, N.A., Punia, R.S., Bowen, M.S., Dolins, S.B., and Clark, T.G. (2012). Tetrahymena Genome Database Wiki: a community-maintained model organism database. Database : the journal of biological databases and curation *2012*, bas007.

Walne, P.R. (1970). Studies on the food value of nineteen genera of algae to juvenile bivalves of the genera Ostrea, Crassostrea, Mercenaria and Mytilus (London,: H.M.S.O.).

Yang, Z. (2007). PAML 4: phylogenetic analysis by maximum likelihood. Molecular biology and evolution *24*, 1586-1591.
